# Supplementary material for: SCGB1D2 inhibits growth of Borrelia burgdorferi and affects susceptibility to Lyme disease
Source: Nat Commun. 2024 Mar 19;15:2041. doi: 10.1038/s41467-024-45983-9 (PMC10950847; doi:10.1038/s41467-024-45983-9)
Supplement: Supplementary file 1 — Supplementary Information [file 41467_2024_45983_MOESM1_ESM.pdf]

Supplementary information for: "*SCGB1D2* inhibits growth of *Borrelia burgdorferi* and affects susceptibility to Lyme disease"

by Satu Strausz, Erik Abner, Grace Blacker, Sarah Galloway, Paige Hansen, Qingying Feng, Brandon T. Lee, Samuel E. Jones, Erin Sanders, Nasa Sinnott-Armstrong, Hele Haapaniemi, Sten Raak, George Ronald Nahass, FinnGen, Estonian Biobank Research Team, Pilleriin Soodla, Urmo Võsa, Tõnu Esko, Irving L. Weissman, Mark Daly, Tuomas Aivelo, Michal Caspi Tal, Hanna M. Ollila

The purpose of this document is to provide information on the cohorts, choices for analytical methods and findings that are not included in the main paper, or where additional details may be useful.

Contents

1. Cohort descriptions
2. Analysis of individual genetic variants
3. Understanding of genetic association
    a. *HLA* locus
    b. *TLR1* locus
    c. *SCGB1D2* locus
4. PheWAS analysis
5. Expression profiling
    a. GTEx
    b. Single cell analysis
6. Functional analyses of *Borrelia burgdorferi*
7. References
8. FinnGen Contributors
9. Estonian Genome Centre Contributors

1. Cohort descriptions

We used two cohorts, FinnGen and Estonian Biobank, which combine genomic data with national health registries.

FinnGen (www.finngen.fi/en) is a joint research project of the public and private sectors, launched in Finland in the autumn of 2017, that aims to genotype 500,000 Finns including prospective and retrospective epidemiological and disease-based cohorts as well as hospital biobank samples[1]. We defined Lyme disease by extracting International Classification of Diseases (ICD)-9 (1048A) and ICD-10 (A69.2) codes from hospital inpatient, hospital outpatient and primary outpatient health registries. Our FinnGen data consisted of 7,354 individuals with Lyme disease and 404,827 controls (Supplementary Table 1).

The Estonian Biobank is a population-based biobank of the Estonian Genome Center at the University of Tartu. Its cohort size is 212,955 participants, which closely reflects the age, sex and geographical distribution of the Estonian population. Lyme disease was based on ICD-10 code A69.2. The Estonian Biobank sample included 18,001 cases and 187,549 disease free controls (Supplementary Table 1).

The resulting full data sample consisted of 617,731 individuals with 25,355 cases and 592,376 controls (Supplementary Table 1).

Supplementary Table 1. *Diagnosis information in individuals with Lyme disease in the study population*

	**Lyme**	**Non-Lyme**
**FinnGen N=412,181**	7,354 (1.8%)	404,827 (98.2%)
**N ICD-10 A69.2**	7,286 (99.1%)	
**N ICD-9 1048A**	68 (0.9%)	
**Sex (male)**	2,760 (37.5%)	179,111 (44.2%)
**Estonian Biobank N=205,550**	18,001 (8.8%)	187,549 (91.2%)
**N ICD-10 A69.2**	18,001 (100%)	
**Sex (male)**	5,078 (28.2%)	65,834 (35.1%)
**Total N=617,731**	25,355 (4.1%)	592,376 (95.9%)

Individuals with and without Lyme disease diagnosis in FinnGen and Estonian Biobank. ICD=International Classification of Diseases.

2. Analysis of individual genetic variants

To study genetics behind Lyme disease we analyzed a total of 617,731 samples from the FinnGen Data Freeze 10 or Estonian Biobank with 25,355 individuals with Lyme disease diagnosis. For the GWAS in both cohorts we performed genome-wide association testing as implemented in the REGENIE [2]. We combined the GWAS results from both cohorts using a fixed-effect meta-analysis model in METAL [3].

The meta-analysis revealed three genome-wide significant signals ($P < 5.0\times10^{-8}$). The characteristics of these loci are presented in Supplementary Table 2. Our analysis pointed to a genome-wide signal in the *TLR1*-locus (rs17616434) and in the HLA-region (rs9276610) located at the HLA class II locus. In addition, we observed the strongest association in *SCGB1D2* where the lead variant was a missense variant rs2232950. Curiously, this variant causes amino acid change from proline to leucine, and this change is predicted deleterious by several databases.

Supplementary Table 2. *Lead variants from meta-analysis for Lyme disease*

CHR	rsid	REF	ALT	Meta-analysis OR [95% CI]	FinnGen OR [95% CI]	EstBB OR [95% CI]
4	rs17616434	T	C	0.92 [0.89-0.94]	0.87 [0.83-0.92]	0.94 [0.91-0.97]
6	rs9276610	A	T	0.92 [0.89-0.94]	0.88 [0.82-0.92]	0.93 [0.90-0.96]
11	rs2232950	C	T	1.21 [1.19-1.23]	1.21 [1.18-1.26]	1.20 [1.17-1.23]

Characterization of three genome-wide significant Lyme disease loci. Effect sizes are reported in terms of alternative allele (ALT). CHR=chromosome, REF=reference allele, OR=odds ratio, CI=confidence interval, EstBB=Estonian Biobank.

3. Understanding of genetic associations

a) *HLA* locus

HLA has a strong and established role in human immune defense. However, its contribution to Lyme disease has not been previously thoroughly explored. Our GWAS results showed a genome-wide significant HLA-locus, and to study this finding in more detail we fine-mapped this region. We computed association statistics with each HLA-allele from the *HLA-A, HLA-B, HLA-C, HLA-DRB1, HLA-DQA1, HLA-DQB1, HLA-DPA1* and *HLA-DPB1* genes and discovered the most significant association with *HLA-DQB1*06:02*.

Similarly, our lead variant rs9276610 was in high linkage disequilibrium (LD) with *HLA-DQB1*06:02* (r^2 = 0.558). As *HLA-DQB1*06:02* is also in high LD with *DRB1*15:01*, we estimated the pairwise LD also for *HLA-DRB1*15:01*. The analysis supported *HLA-DQB1*06:02* as the most significant HLA-allele to associate with Lyme disease (Supplementary Table 3-4).

Supplementary Table 3. *Linkage disequilibrium (r2) between lead variants from FinnGen and Meta-analysis and the HLA-alleles in FinnGen.*

**HLA-allele**	**r^2 with rs9273375 / FinnGen**	**r^2 with rs9276610 / Meta-analysis**
*DQB1*06:02*	0.901	0.558
*DRB1*15:01*	0.885	0.550
*DRB5*01:01*	0.887	0.549
*DQA1*01:02*	0.640	0.402

Supplementary Table 4. *Linkage disequilibrium (r2) between DRB1*15:01 and DQB1*06:02 in FinnGen.*

**HLA-allele**	**HLA-allele**	**r^2**
*DRB1*15:01*	*DQB1*06:02*	0.986

Supplementary Table 5. *HLA allele associations with Lyme disease in FinnGen*

**HLA-allele**	**beta**	**se**	**Unadjusted P-value**
*DQB1*06:02*	0.173	0.0262	4.413E-11
*DRB5*01:01*	0.171	0.0262	5.931E-11
*DRB1*15:01*	0.171	0.0262	6.262E-11
*DQA1*01:02*	0.135	0.0247	4.448E-8
*B*07:02*	0.074	0.027	5.86E-3
*B*08:01*	-0.083	0.032	8.546E-3
*C*07:02*	0.066	0.026	1.167E-2
*DQB1*02:02*	-0.104	0.046	2.290E-2

We show associations with P-value < 0.05. The tests were computed with two-sided logistic regression.

Supplementary Table 6. *HLA allele associations with Lyme disease in Estonian Biobank*

**HLA-allele**	**beta**	**se**	**Unadjusted P-value**
*B*56:01*	-0.6132	0.165	0.0002
*A*25:01*	0.3299	0.128	0.0100
*C*01:02*	-0.2672	0.103	0.0092
*DRB1*04:01*	-0.2437	0.103	0.0184
*A*03:01*	-0.1522	0.067	0.0221

We show associations with P-value < 0.05. The tests were computed with two-sided logistic regression.

Supplementary Table 7. *HLA amino acid associations with Lyme disease in FinnGen*

HLA amino acid	beta	se	Unadjusted P-value
DRB1 P0S	0.173	0.026	3.66E-11
DRB1 P27A	0.173	0.026	3.66E-11
DRB1 P11P	0.160	0.026	5.617E-11
DRB1 P13R	0.160	0.026	5.617E-10
DRB1 P134L	0.160	0.026	5.617E-10
DRB1 P143M	0.160	0.026	5.617E-10
DRB1 P97Q	0.144	0.026	1.641E-8
DQA1 P207M	0.136	0.025	3.848E-8
DQB1 P9F	0.123	0.024	2.717E-7
DQB1 P87F	0.191	0.024	2.282E-5
DQB1 P125G	0.092	0.024	1.134E-4
DQB1 P-4L	0.090	0.024	1.386E-4
DQB1 P9Y	-0.168	0.048	3.950E-4

We show associations with P-value < 0.001. The tests were computed with two-sided logistic regression.

Supplementary Figure 1. Regional association at the HLA locus.

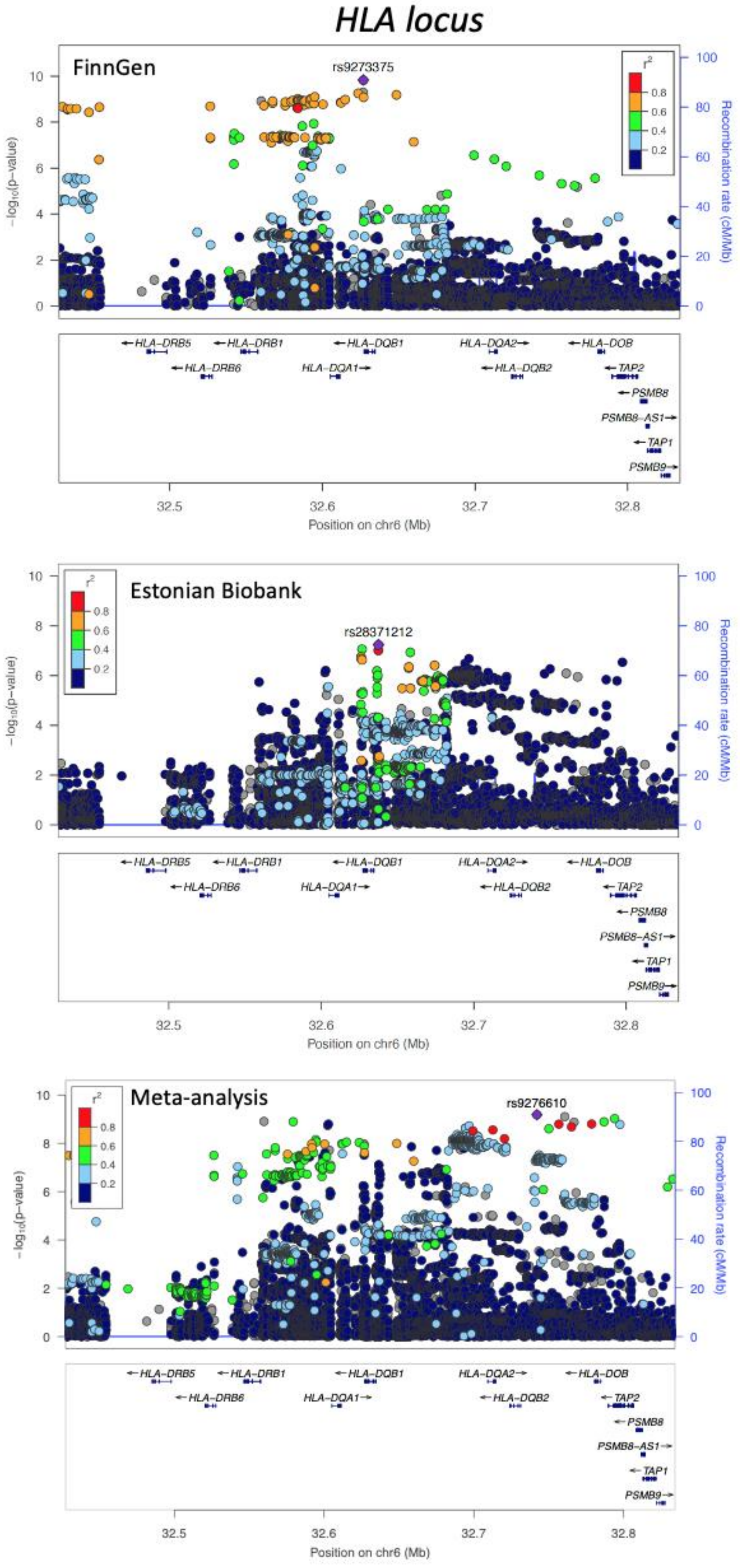

Supplementary Figure 2. *Meta-analysis of HLA locus conditioning for main effect.*

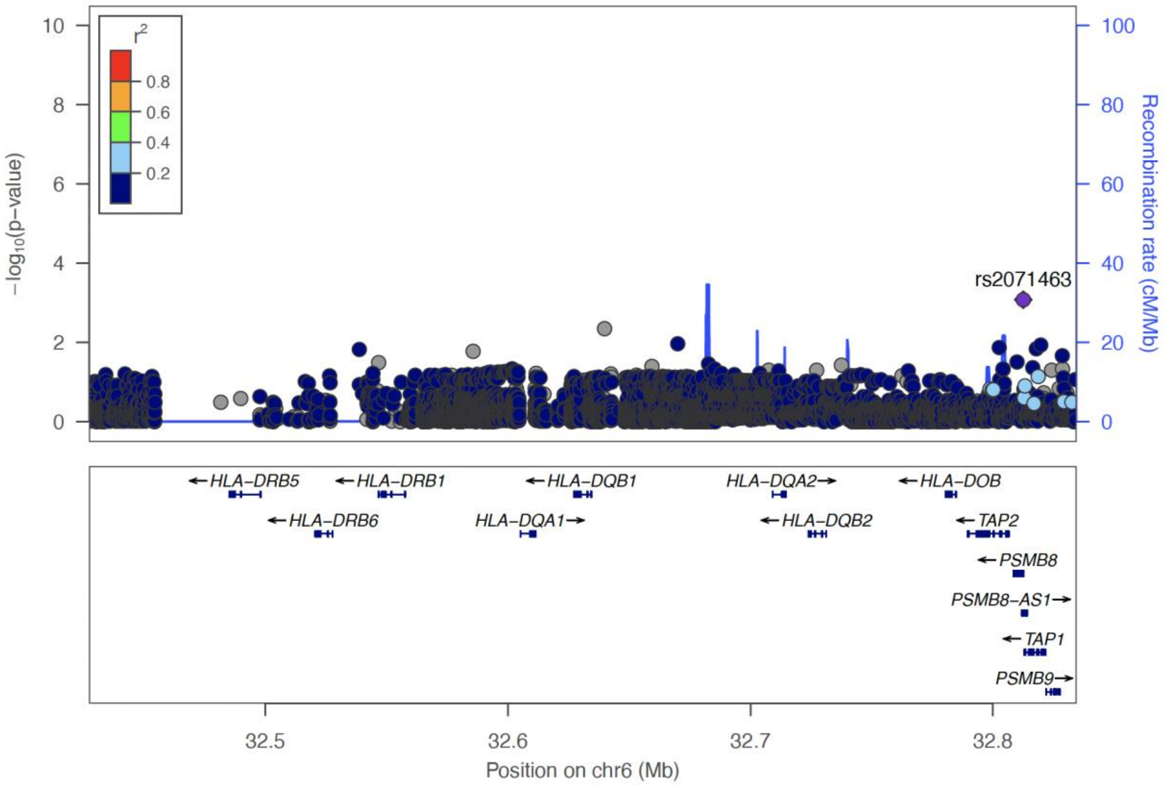

b) *TLR1* locus

Toll-like receptors control innate immune responses that affect the first line defense against pathogens and infections. The TLR locus that associates with Lyme disease has been previously associated in particular with TLR activation and following cytokine response [4]. In our study we identified a signal with a non-coding variant at the TLR1 locus that was in high LD with two missense variants at TLR1 (rs5743618, Ser602Ile, r2 = 0.90; and rs4833095, Asn248Ser, r2 = 0.89). Conditional analysis adjusting for the main effect (rs17616434) did not reveal additional signals. The P-values for missense variants after conditioning for the main effect was (rs5743618, Ser602Ile, P = 0.22; and rs4833095, Asn248Ser, P = 0.40) suggesting that the signal from these missense variants is either captured or shared with the lead variant. Finally, Supplementary Figure 3 shows robustness of the signal across both cohorts.

Supplementary Figure 3. Regional association at the *TLR1* locus in FinnGen, Estonian Biobank and in the meta-analysis.

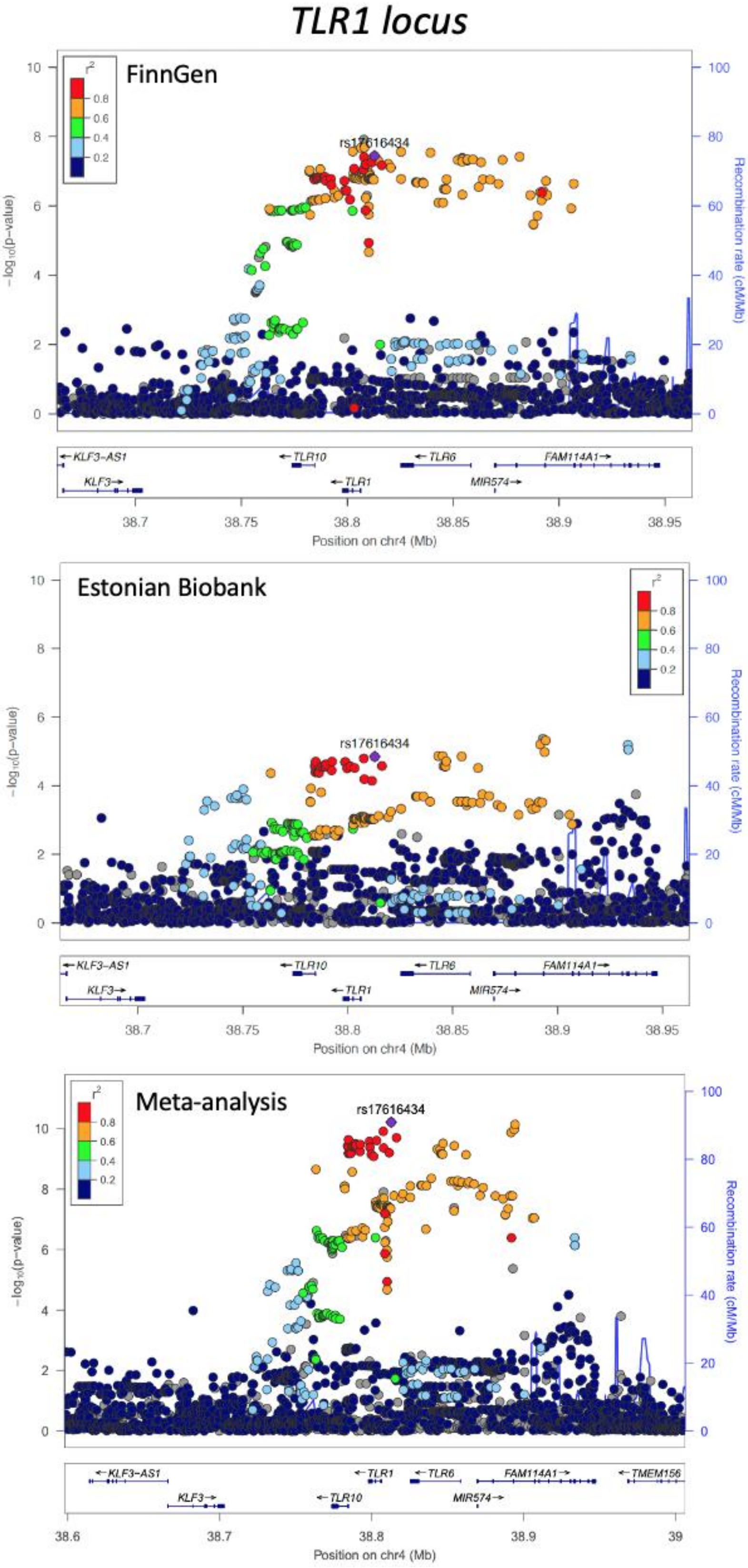

c) *SCGB1D2* locus

Our meta-analysis showed a novel genome-wide significant finding related to Lyme disease in chromosome 11 (rs2232950). This causes amino acid change from proline to leucine indicating a deleterious mutation by several algorithms[5,6].

Supplementary Figure 4. Regional association at the SCGB1D2 locus

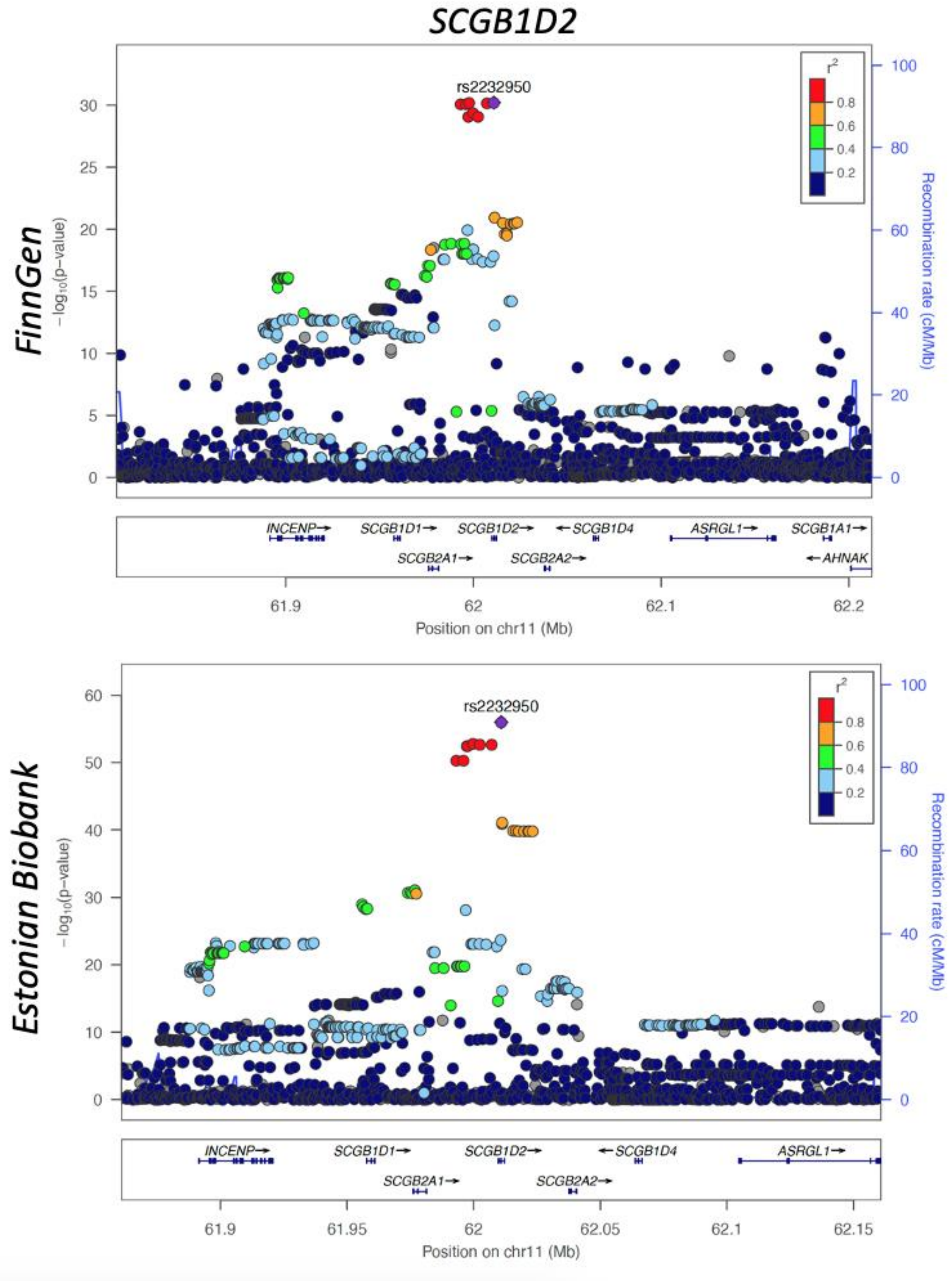

To examine *SCGB1D2* locus and its genomic variation's causality to Lyme disease in more detail, we fine-mapped this region utilizing the "Sum of Single Effects" -model, called *SuSiE* [7]. The credible set included six variants including the common missense variant rs2232950. Furthermore, *SuSiE* predicted posterior probability of 0.22 to the lead variant rs2232950.

Similarly to FinnGen, the locus in EstBB contains several significant variants. Fine-mapping the locus with HyprColoc [8] revealed all the SNPs to be in high LD with each other, and conditional analysis suggested the lead SNP rs2232950 was the likely causal variant (Supplementary Figure 5).

Supplementary Figure 5. Conditional analysis in SCGB1D2 locus. Adjusting for rs2232950 removes association signal at the SCGB1D2 locus.

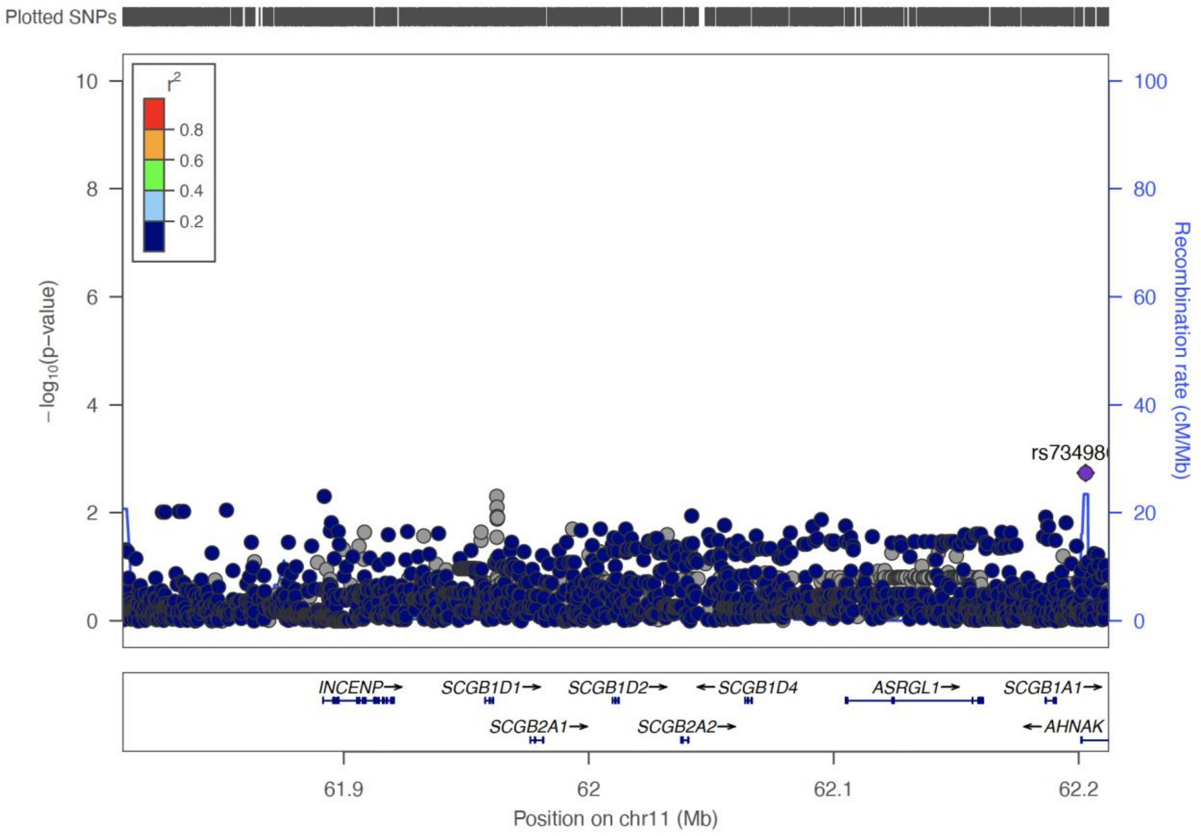

The figure depicts P-value (-log10 scale) plot of colocalization of chr11-wide association study with Lyme disease as a binary trait (conditional for rs2232950). Each dot represents a single nucleotide variant (SNV). The highlighted SNV (mahogany red triangle) is the candidate causal SNV. Colors filled in the dots depict its linkage disequilibrium with the candidate causal SNV. No independent chr11-wide significance was observed for other SNVs.

Prolines are often the initiators of α-helix structures [9]. Pro53 is the first residue in SCGB1D2 H3-helix backbone, therefore a Pro>Leu substitution at this position will likely destabilize the α-helical structure, as the downstream amino acids (Val56 and Ala57) will lose an anchor-point (Supplementary Figure 6).

Supplementary Figure 6. Overall structure of Lipophilin B protein dimer and evaluation utilizing AlphaFold2 structure prediction.

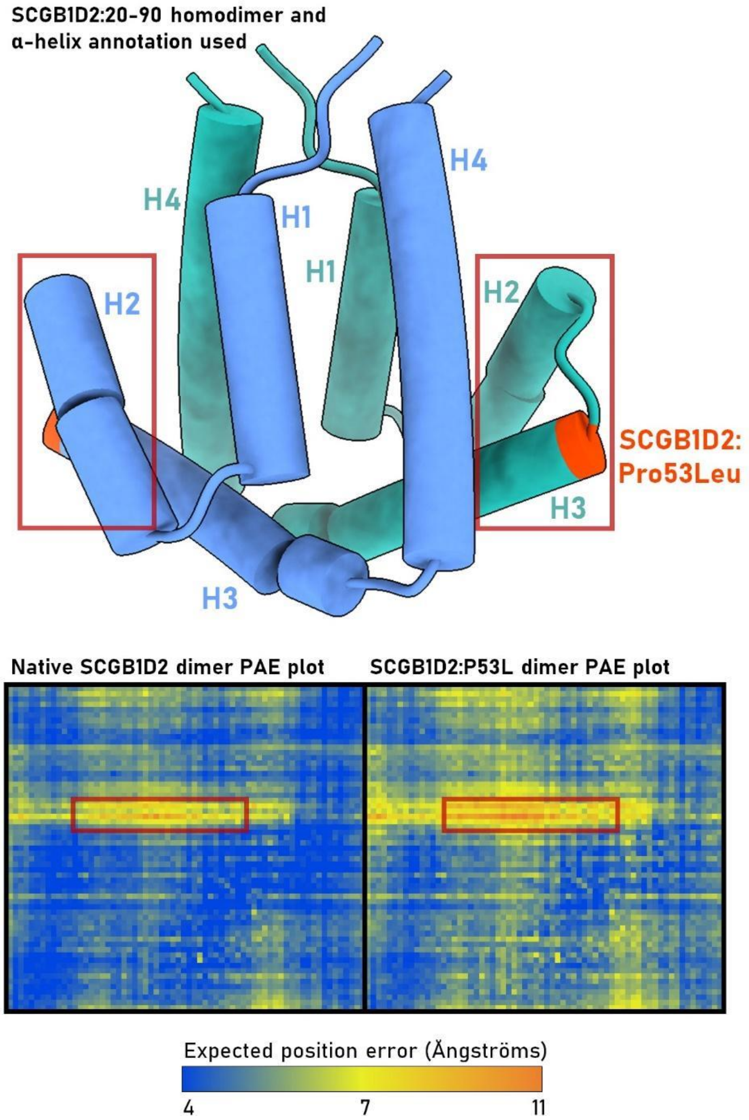

Upper figure depicts the homodimerized form of SCGB1D2, as predicted by AlphaFold2 (best model). Helix nomenclature has been annotated. SCGB1D2:Pro53Leu has been colored in orange on both dimers. Heatmaps below depict the predicted aligned error (PAE) plots created by AlphaFold2. Comparing dimeric structures of SCGB1D2 and the mutant SCGB1D2:Pro53Leu, we witness an increase in the PAE of the complete structure (increase in yellow-red color scheme), especially around the H2-H3 helices (red squares). Presumably, this instability decreases the stability of the whole complex, thereby decreasing the affinity towards natural ligands of SCGB1D2 protein complexes. Only amino acids of the full SCGB1D2 protein 20-90 were used for generating this data. On the dimer model in the lower right, the Pro53Leu point mutation location has been highlighted in orange. Molecular graphics and analyses performed with UCSF ChimeraX (version 1.5rc202211080803), developed by the Resource for Biocomputing, Visualization, and Informatics at the University of California, San Francisco, with support from National Institutes of Health R01-GM129325 and the Office of Cyber Infrastructure and Computational Biology, National Institute of Allergy and Infectious Diseases [10]**.**

4. PheWAS analysis

We performed a phenome-wide association analysis (PheWAS) to explore the association between the missense variant rs2232950 and 2,202 disease endpoints from FinnGen. FinnGen endpoints include primarily electronic health record derived phenotypes. To complement this analysis, we computed PheWAS also using the OpenTargets platform, which includes traits from publicly available GWASes and traits from other biobanks. This analysis did not reveal additional significant associations besides its association with Lyme disease in FinnGen and rs2232950. Therefore, this analysis did not provide additional insight into the function of *SCGB1D2* (Supplementary Figure 7).

Supplementary Figure 7. Open targets PheWAS with rs2232950

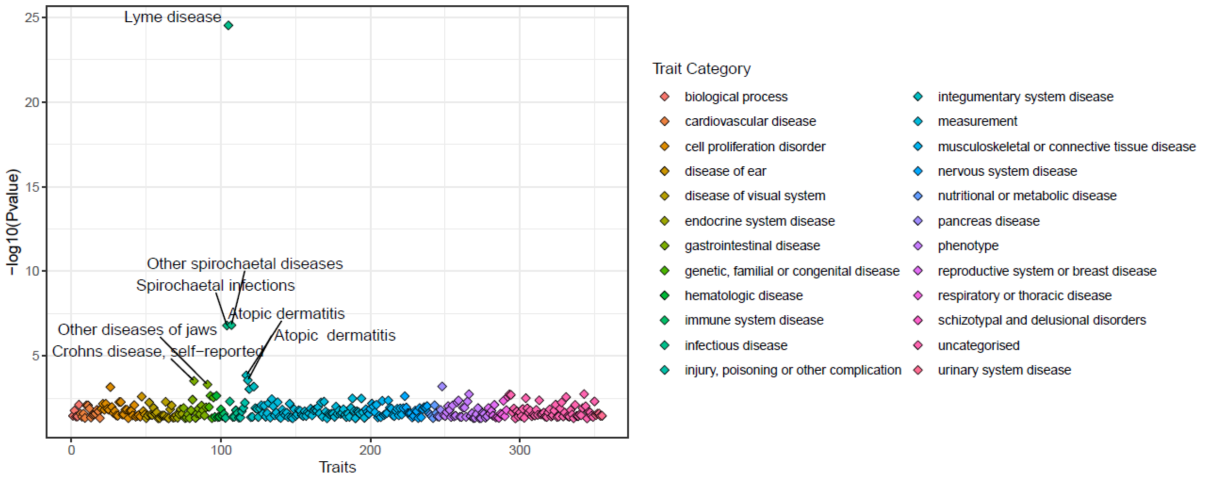

Phenome-wide association (PheWAS) from publicly available data from the OpenTargets platform are visualized by trait and their -log10(P-values) in the y-axis. Colors represent trait categories.

Furthermore, to elucidate the possible broader association of SCGB1D2 Pro53Leu with pathogens we analyzed association across 36 different disease categories in EstBB, including tick-mediated diseases (tick-borne encephalitis), general arthropod behavioral markers (scabies), spirochaete bacterium phylum members (syphilis) and other bacterial diseases (e.g. sepsis, scarlet fever and erysipelas) of which none were associated with Lyme disease SCGB1D2 Pro53Leu variant (Supplementary Figure 8).

Supplementary Figure 8. Results of a single variant association study with various infectious agent categories

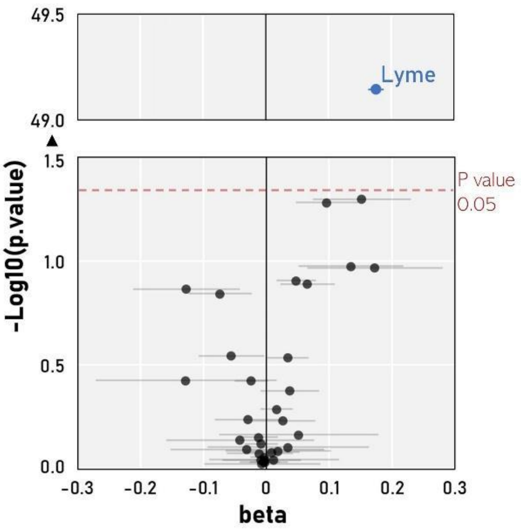

ICD-10	n_EstBB	Cohort description	-Log10P	beta	SE
A69	17,942	Other spirochaetal infections	49.143	0.176	0.012
A26	367	Erysipeloid	1.297	0.152	0.078
A54	900	Gonococcal infection	1.28	0.097	0.05
A84	698	Tick-borne encephalitis	1.278	0.11	0.056
A53	322	Other and unspecified syphilis	0.975	0.135	0.083
A40	192	Streptococcal septicaemia	0.968	0.173	0.108
A74	2,341	Other diseases caused by chlamydiae	0.906	0.048	0.031
B95	1,211	Streptococcus and staphylococcus as the cause of diseases classified to other chapters	0.89	0.065	0.043
A36	322	Diphtheria	0.865	-0.127	0.085
A16	892	Respiratory tuberculosis, not confirmed bacteriologically or histologically	0.843	-0.073	0.05
A03	832	Shigellosis	0.545	-0.055	0.052
A41	2,139	Other septicaemia	0.535	0.035	0.033
A28	106	Other zoonotic bacterial diseases, not elsewhere classified	0.425	-0.128	0.144
A49	3,250	Bacterial infection of unspecified site	0.424	-0.023	0.026
B88	1,034	Other infestations	0.376	0.037	0.046
B96	3,463	Other bacterial agents as the cause of diseases classified to other chapters	0.288	0.017	0.026
A50-A53	550	Syphilis custom	0.285	0.043	0.067
A31	820	Infection due to other mycobacteria	0.238	-0.029	0.052
A15	892	Respiratory tuberculosis, bacteriologically and histologically confirmed	0.231	0.027	0.05
B89	135	Unspecified parasitic disease	0.165	0.052	0.127
A04	2,440	Other bacterial intestinal infections	0.152	-0.012	0.03
A18	161	Tuberculosis of other organs	0.139	-0.041	0.118
A38	3,295	Scarlet fever	0.121	-0.008	0.026
A01	137	Typhoid and paratyphoid fevers	0.104	0.035	0.129
A00	150	Cholera	0.094	-0.03	0.122
A48	314	Other bacterial diseases, not elsewhere classified	0.086	0.019	0.084
B86	6,021	Scabies	0.083	-0.004	0.02
A66	1,145	Yaws	0.081	0.009	0.044
A02	804	Other salmonella infections	0.074	-0.01	0.053
A46	5,079	Erysipelas	0.056	-0.003	0.021
A05	208	Other bacterial foodborne intoxications, not elsewhere classified	0.044	0.012	0.103
A42	574	Actinomycosis	0.044	-0.008	0.063
A56	9,222	Other sexually transmitted chlamydial diseases	0.043	-0.002	0.016
B90	1,586	Sequelae of tuberculosis	0.035	-0.004	0.038
A37	4,119	Whooping cough	0.033	-0.002	0.024
A51	258	Early syphilis	0.025	-0.006	0.092

36 different infectious disease categories (ICD-10 classification system) were used to create study cohorts within EstBB, using electronic health records from the Estonian medical system between 2004-2021. Genetic relationship matrices and logistic regression for (rs2232950) SCGB1D2:Pro53Leu for each cohort was calculated with Rerenie[8]. Plot on left displays associations according to their P value (-log10 scale) and beta coefficient values. Lead hit for A69 category ("other spirochaetal infections", of which A69.2 "Lyme disease" makes ~97%) is marked in blue. The red horizontal line represents a threshold of statistical significance (P = 0.05). Table on the right displays the ICD-10 categories, cohort description, P value, effect size, standard error and is sorted according to P-value (-log10 scale).

5. Expression profiling

a) GTEx

We examined RNA expression across tissue types using The Genotype-Tissue Expression (GTEx) v8 using the fully processed and normalized gene expression matrices for each tissue. These data contain RNA expression samples from 948 donors across 54 tissues. These same values are used for eQTL calculations by GTEx. We extracted the values for *SCGB1D2* in these data and plotted the normalized values per tissue (Supplementary Figure 9). The two skin types (sun exposed and unexposed) show the highest expression levels of *SCBG1D2*.

Supplementary Figure 9. Tissue distribution of *SCGB1D2* expression

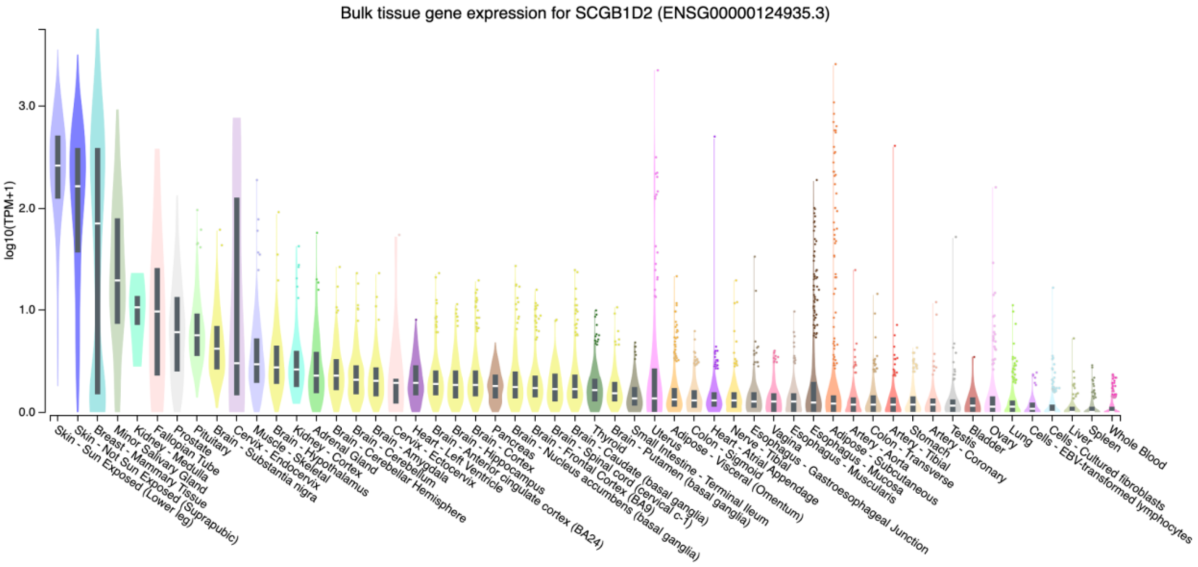

Expression pattern of SCGB1D2 across human tissues. We obtained RNA expression data from the GTEx project[11] and examined the expression profile of SCGB1D2 across tissues. A total of 701 individuals with expression values showed the highest expression in the skin (sun exposed) and skin (not sun exposed).

b) Single cell analysis

In order to understand the relevant cell types for *SCGB1D2* expression from the skin we examined single cell sequencing data from skin. We observed that *SCGB1D2* was predominantly expressed by the sweat gland cells (Supplementary Figure 10).

Supplementary Figure 10. Expression of *SCGB1D2* by cell type in the skin

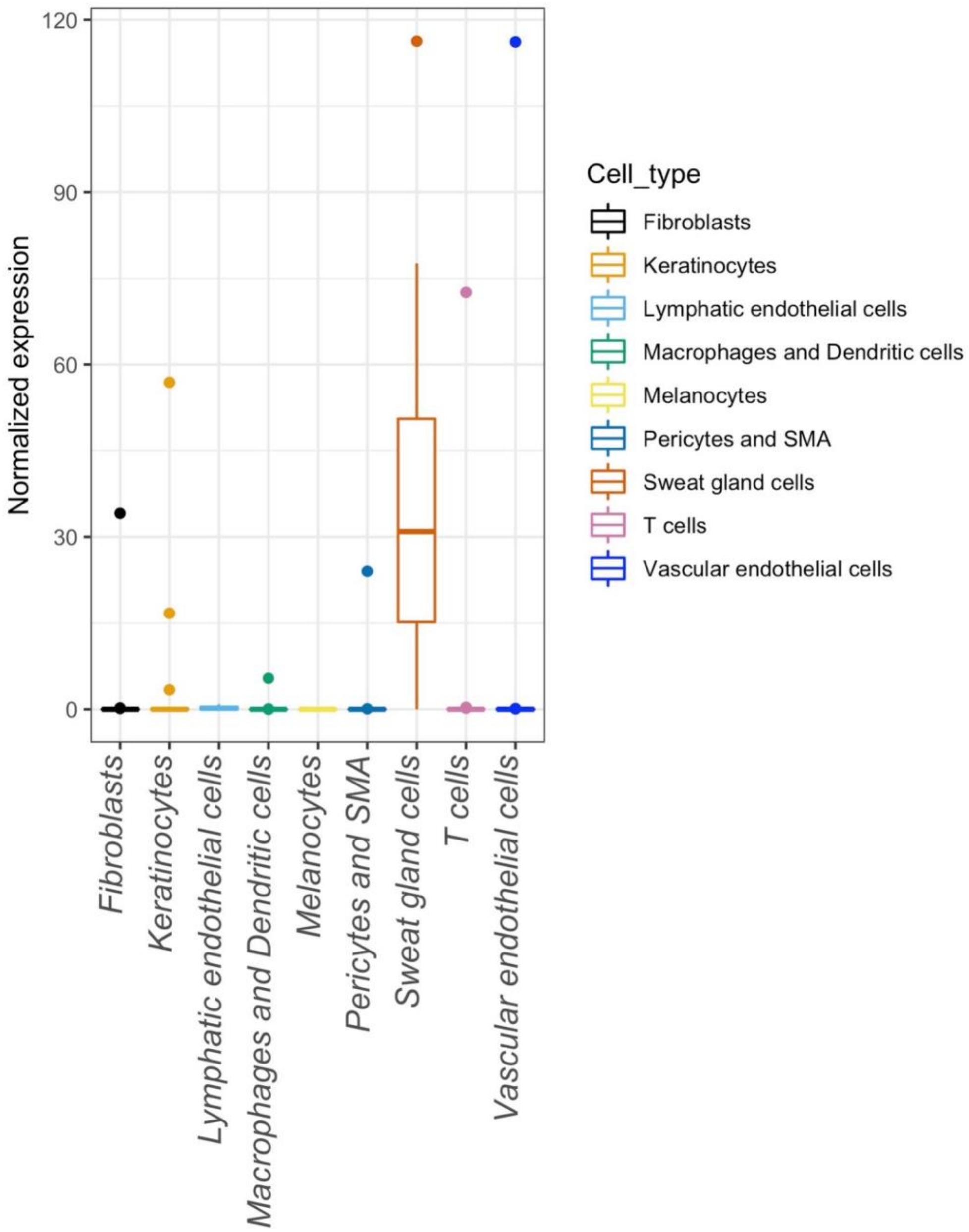

Single cell sequencing data shows SCGB1D2 expression is specific for sweat gland cells. Data from He et al [12].

6. Functional analyses of *Borrelia burgdorferi*

The results below support our main findings in live *Bb*. We estimate the effect of *SCGB1D2* P53L on *Bb* growth and examine the killing capability of *SCGB1D2* recombinant protein on live *Bb* (Supplementary Figure 11-14).

Supplementary Figure 11. *Borrelia burgdorferi* growth inhibition by *SCGB1D2* P53L

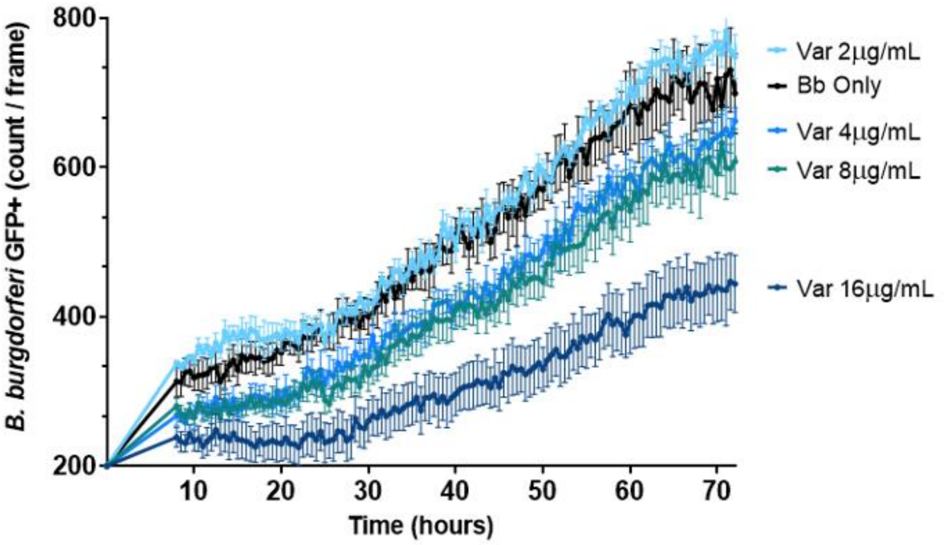

Timescale analysis of Borrelia burgdorferi growth inhibition by SCGB1D2 P53L *over 72h hours. Y-axis represents* green fluorescent protein (GFP) *count per frame and X-axis represents time. Concentrations tested are 2 to 16 µg/mL.*

Supplementary Figure 12. *Borrelia burgdorferi* killing by *SCGB1D2*

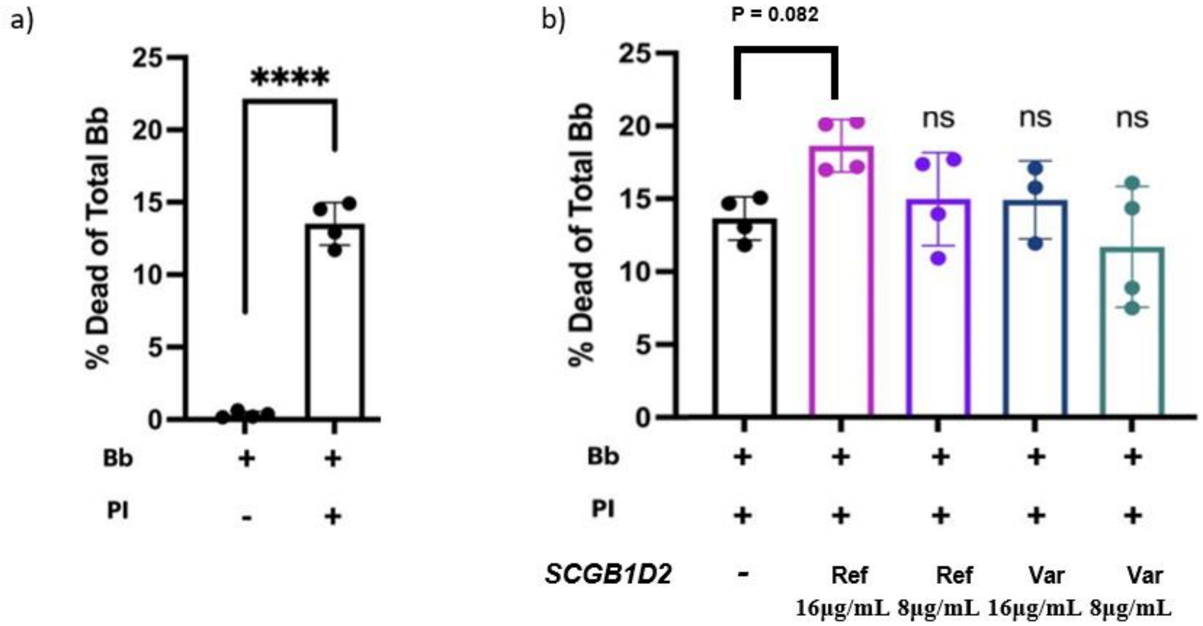

*Borrelia burgdorferi (Bb) spirochetes expressing green fluorescent protein (GFP) were incubated with a) with or without propidium iodide (PI). b) either 8µg/mL or 16µg/mL of reference (Ref) or variant (Var) SCGB1D2 protein in the presence of PI to measure Bb death by SCGB1D2. After 24 hours of incubation, an aliquot of each culture was analyzed by flow cytometry for loss of GFP. Overall ANOVA was used comparing Bb with PI and Bb with PI and SCGB1D2 proteins (ANOVA F(4, 14)=3.185, P=0.0467). ****P<0.0001; ns, not significant.*

Supplementary Figure 13. *Gating scheme to distinguish live and dead bacteria*

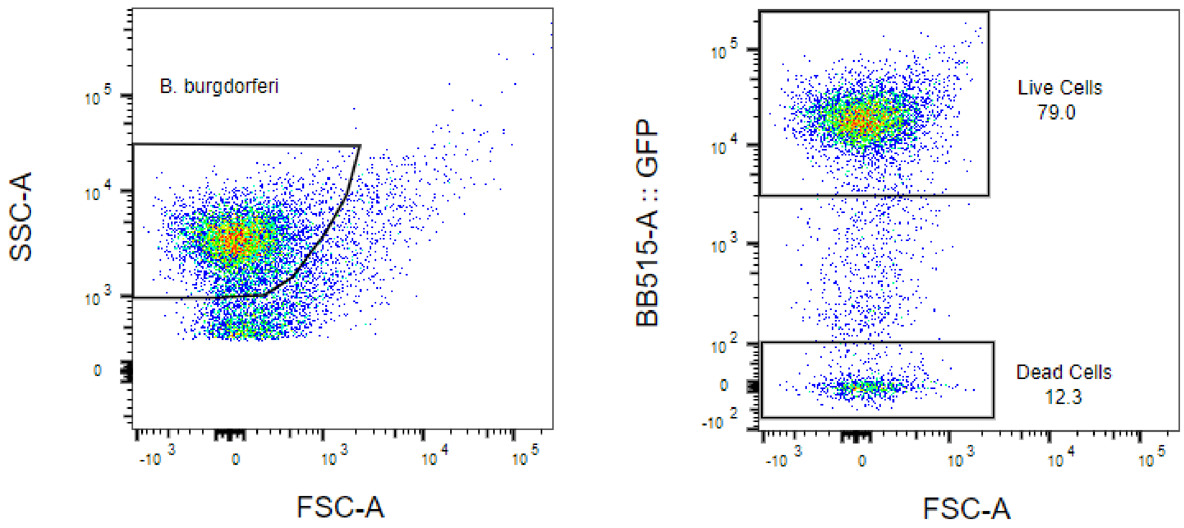

Gating scheme is shown for green fluorescent protein (GFP) positive and GFP negative Borrelia Burgdorferi (Bb) to distinguish live and dead bacteria after 24 hours in BSK-H culture media and exposed to varying levels of SCGB1D2 protein in a Bb growth inhibition assay, with the addition of 1.5µL propidium iodide (Millipore Sigma) per well prior to incubation. After 24 hours of incubation, samples were fixed in 4% paraformaldehyde, resuspended in flow cytometry buffer (2% FBS, 1mmol EDTA, in PBS), and analyzed by flow cytometry for presence of GFP.

Supplementary Figure 14. In vivo imaging system (IVIS) quantification of SCGB1D2 prophylaxic effect on intradermal infection with N40D10/E9 *Bb* at Day 0

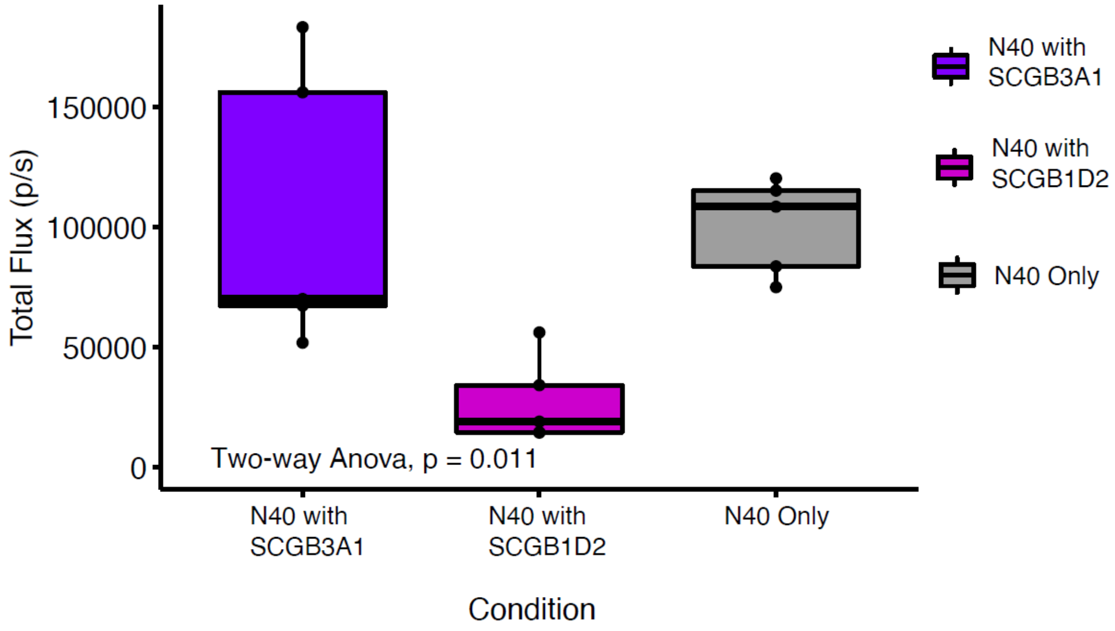

IVIS imaging of 1 min exposures of mice at day 0 post-infection of Bb(N40D10/E9) where the Bb had been co-incubated co-infected with SCGB1D2 or SCGB3A1 or no protein control. Total flux (p/s) signals were quantified by gating individual mice at the injection site 15 min after being injected with 277 mg/kg sterile filtered D-luciferin dissolved in phosphate buffered saline (PBS).

7. References

1 Kurki, M. I. *et al.* FinnGen provides genetic insights from a well-phenotyped isolated population. *Nature* **613**, 508-518, doi:10.1038/s41586-022-05473-8 (2023).

2 Mbatchou, J. *et al.* Computationally efficient whole-genome regression for quantitative and binary traits. *Nat Genet* **53**, 1097-1103, doi:10.1038/s41588-021-00870-7 (2021).

3 Willer, C. J., Li, Y. & Abecasis, G. R. METAL: fast and efficient meta-analysis of genomewide association scans. *Bioinformatics* **26**, 2190-2191, doi:10.1093/bioinformatics/btq340 (2010).

4 Mikacenic, C., Reiner, A. P., Holden, T. D., Nickerson, D. A. & Wurfel, M. M. Variation in the TLR10/TLR1/TLR6 locus is the major genetic determinant of interindividual difference in TLR1/2-mediated responses. *Genes Immun* **14**, 52-57, doi:10.1038/gene.2012.53 (2013).

5 Adzhubei, I. A. *et al.* A method and server for predicting damaging missense mutations. *Nat Methods* **7**, 248-249, doi:10.1038/nmeth0410-248 (2010).

6 Ng, P. C. & Henikoff, S. SIFT: Predicting amino acid changes that affect protein function. *Nucleic Acids Res* **31**, 3812-3814, doi:10.1093/nar/gkg509 (2003).

7 Wang G, S. A., Carbonetto P, Stephens M. A Simple New Approach to Variable Selection in Regression, with Application to Genetic Fine Mapping. *Journal of the Royal Statistical Society: Series B (Statistical Methodology).* **82**, 1273–1300 (2020).

8 Foley, C. N. *et al.* A fast and efficient colocalization algorithm for identifying shared genetic risk factors across multiple traits. *Nat Commun* **12**, 764, doi:10.1038/s41467-020-20885-8 (2021).

9 Woolfson, D. N. & Williams, D. H. The influence of proline residues on alpha-helical structure. *FEBS Lett* **277**, 185-188, doi:10.1016/0014-5793(90)80839-b (1990).

10 Pettersen, E. F. *et al.* UCSF ChimeraX: Structure visualization for researchers, educators, and developers. *Protein Sci* **30**, 70-82, doi:10.1002/pro.3943 (2021).

11 Consortium, G. T. The Genotype-Tissue Expression (GTEx) project. *Nat Genet* **45**, 580-585, doi:10.1038/ng.2653 (2013).

12 He, H. *et al.* Single-cell transcriptome analysis of human skin identifies novel fibroblast subpopulation and enrichment of immune subsets in atopic dermatitis. *J Allergy Clin Immunol* **145**, 1615-1628, doi:10.1016/j.jaci.2020.01.042 (2020).

8. FinnGen Contributors

Full Name	Affiliation	E-mail	Role 1	Role 2
Aarno Palotie	Institute for Molecular Medicine Finland (FIMM), HiLIFE, University of Helsinki, Helsinki, Finland; Broad Institute of MIT and Harvard; Massachusetts General Hospital	aarno.palotie@helsinki.fi	**Steering Committee**	**Steering Committee**
Mark Daly	Institute for Molecular Medicine Finland (FIMM), HiLIFE, University of Helsinki, Helsinki, Finland; Broad Institute of MIT and Harvard; Massachusetts General Hospital	mark.daly@helsinki.fi	**Steering Committee**	**Steering Committee**
Bridget Riley-Gills	Abbvie, Chicago, IL, United States	bridget.rileygillis@abbvie.com	**Steering Committee**	**Pharmaceutical companies**
Howard Jacob	Abbvie, Chicago, IL, United States	howard.jacob@abbvie.com	**Steering Committee**	**Pharmaceutical companies**
Dirk Paul	Astra Zeneca, Cambridge, United Kingdom	dirk.paul@astrazeneca.com	**Steering Committee**	**Pharmaceutical companies**
Slavé Petrovski	Astra Zeneca, Cambridge, United Kingdom	slav.petrovski@astrazeneca.com	**Steering Committee**	**Pharmaceutical companies**
Chia-Yen Chen	Biogen, Cambridge, MA, United States	chiayen.chen@biogen.com	**Steering Committee**	**Pharmaceutical companies**
Sally John	Biogen, Cambridge, MA, United States	sally.john@biogen.com	**Steering Committee**	**Pharmaceutical companies**
George Okafo	Boehringer Ingelheim, Ingelheim am Rhein, Germany	george.okafo@boehringer-ingelheim.com	**Steering Committee**	**Pharmaceutical companies**
Robert Plenge	Bristol Myers Squibb, New York, NY, United States	robert.plenge@bms.com	**Steering Committee**	**Pharmaceutical companies**
Joseph Maranville	Bristol Myers Squibb, New York, NY, United States	joseph.maranville@bms.com	**Steering Committee**	**Pharmaceutical companies**
Mark McCarthy	Genentech, San Francisco, CA, United States	mccarthy.mark@gene.com	**Steering Committee**	**Pharmaceutical companies**
Rion Pendergrass	Genentech, San Francisco, CA, United States	penders2@gene.com	**Steering Committee**	**Pharmaceutical companies**
Margaret G. Ehm	GlaxoSmithKline, Collegeville, PA, United States	meg.g.ehm@gsk.com	**Steering Committee**	**Pharmaceutical companies**
Kirsi Auro	GlaxoSmithKline, Espoo, Finland	kirsi.m.auro@gsk.com	**Steering Committee**	**Pharmaceutical companies**
Simonne Longerich	Merck, Kenilworth, NJ, United States	simonne.longerich@merck.com	**Steering Committee**	**Pharmaceutical companies**
Anders Mälarstig	Pfizer, New York, NY, United States	anders.malarstig@pfizer.com	**Steering Committee**	**Pharmaceutical companies**
Anna Vlahiotis	Pfizer, New York, NY, United States	anna.vlahiotis@pfizer.com	**Steering Committee**	**Pharmaceutical companies**
Katherine Klinger	Translational Sciences, Sanofi R&D, Framingham, MA, USA	katherine.klinger@sanofi.com	**Steering Committee**	**Pharmaceutical companies**
Clement Chatelain	Translational Sciences, Sanofi R&D, Framingham, MA, USA	clement.chatelain@sanofi.com	**Steering Committee**	**Pharmaceutical companies**
Matthias Gossel	Translational Sciences, Sanofi R&D, Framingham, MA, USA	matthias.gossel@sanofi.com	**Steering Committee**	**Pharmaceutical companies**
Karol Estrada	Maze Therapeutics, San Francisco, CA, United States	kestrada@mazetx.com	**Steering Committee**	**Pharmaceutical companies**

Robert Graham	Maze Therapeutics, San Francisco, CA, United States	rgraham@mazetx.com	**Steering Committee**	**Pharmaceutical companies**
Dawn Waterworth	Janssen Research & Development, LLC, Spring House, PA, United States	dwaterwo@its.jnj.com	**Steering Committee**	**Pharmaceutical companies**
Chris O´Donnell	Novartis Institutes for BioMedical Research, Cambridge, MA, United States	chris.odonnell@novartis.com	**Steering Committee**	**Pharmaceutical companies**
Nicole Renaud	Novartis Institutes for BioMedical Research, Cambridge, MA, United States	nicole.renaud@novartis.com	**Steering Committee**	**Pharmaceutical companies**
Tomi P. Mäkelä	HiLIFE, University of Helsinki, Finland, Finland	tomi.makela@helsinki.fi	**Steering Committee**	**University of Helsinki & Biobanks**
Jaakko Kaprio	Institute for Molecular Medicine Finland (FIMM), HiLIFE, University of Helsinki, Helsinki, Finland	jaakko.kaprio@helsinki.fi	**Steering Committee**	**University of Helsinki & Biobanks**
Minna Ruddock	Arctic biobank / University of Oulu	minna.ruddock@oulu.fi	**Steering Committee**	**University of Helsinki & Biobanks**
Petri Virolainen	Auria Biobank / University of Turku / Hospital District of Southwest Finland, Turku, Finland	petri.virolainen@tyks.fi	**Steering Committee**	**University of Helsinki & Biobanks**
Antti Hakanen	Auria Biobank / University of Turku / Hospital District of Southwest Finland, Turku, Finland	antti.hakanen@tyks.fi	**Steering Committee**	**University of Helsinki & Biobanks**
Terhi Kilpi	THL Biobank / Finnish Institute for Health and Welfare (THL), Helsinki, Finland	terhi.kilpi@thl.fi	**Steering Committee**	**University of Helsinki & Biobanks**
Markus Perola	THL Biobank / Finnish Institute for Health and Welfare (THL), Helsinki, Finland	markus.perola@thl.fi	**Steering Committee**	**University of Helsinki & Biobanks**
Jukka Partanen	Finnish Red Cross Blood Service / Finnish Hematology Registry and Clinical Biobank, Helsinki, Finland	jukka.partanen@veripalvelu.fi	**Steering Committee**	**University of Helsinki & Biobanks**
Taneli Raivio	Helsinki Biobank / Helsinki University and Hospital District of Helsinki and Uusimaa, Helsinki	taneli.raivio@hus.fi	**Steering Committee**	**University of Helsinki & Biobanks**
Jani Tikkanen	Northern Finland Biobank Borealis / University of Oulu / Northern Ostrobothnia Hospital District, Oulu, Finland	jani.tikkanen@ppshp.fi	**Steering Committee**	**University of Helsinki & Biobanks**
Raisa Serpi	Northern Finland Biobank Borealis / University of Oulu / Northern Ostrobothnia Hospital District, Oulu, Finland	raisa.serpi@ppshp.fi	**Steering Committee**	**University of Helsinki & Biobanks**
Tarja Laitinen	Finnish Clinical Biobank Tampere / University of Tampere / Pirkanmaa Hospital District, Tampere, Finland	tarja.laitinen@pshp.fi	**Steering Committee**	**University of Helsinki & Biobanks**
Veli-Matti Kosma	Biobank of Eastern Finland / University of Eastern Finland / Northern Savo Hospital District, Kuopio, Finland	veli-matti.kosma@uef.fi	**Steering Committee**	**University of Helsinki & Biobanks**
Jari Laukkanen	Central Finland Biobank / University of Jyväskylä / Central Finland Health Care District, Jyväskylä, Finland	jari.laukkanen@ksshp.fi	**Steering Committee**	**University of Helsinki & Biobanks**
Marco Hautalahti	FINBB - Finnish biobank cooperative	marco.hautalahti@finbb.fi	**Steering Committee**	**University of Helsinki & Biobanks**
Outi Tuovila	Business Finland, Helsinki, Finland	outi.tuovila@businessfinland.fi	**Steering Committee**	**Other Experts/ Non-Voting Members**
Raimo Pakkanen	Business Finland, Helsinki, Finland	raimo.pakkanen@businessfinland.fi	**Steering Committee**	**Other Experts/ Non-Voting Members**
Jeffrey Waring	Abbvie, Chicago, IL, United States	jeff.waring@abbvie.com	**Scientific Committee**	**Pharmaceutical companies**
Bridget Riley-Gillis	Abbvie, Chicago, IL, United States	bridget.rileygillis@abbvie.com	**Scientific Committee**	**Pharmaceutical companies**

Fedik Rahimov	Abbvie, Chicago, IL, United States	fedik.rahimov@abbvie.com	**Scientific Committee**	**Pharmaceutical companies**
Ioanna Tachmazidou	Astra Zeneca, Cambridge, United Kingdom	ioanna.tachmazidou@astrazeneca.com	**Scientific Committee**	**Pharmaceutical companies**
Chia-Yen Chen	Biogen, Cambridge, MA, United States	chiayen.chen@biogen.com	**Scientific Committee**	**Pharmaceutical companies**
Zhihao Ding	Boehringer Ingelheim, Ingelheim am Rhein, Germany	zhihao.ding@boehringer-ingelheim.com	**Scientific Committee**	**Pharmaceutical companies**
Marc Jung	Boehringer Ingelheim, Ingelheim am Rhein, Germany	marc_oliver.jung@boehringer-ingelheim.com	**Scientific Committee**	**Pharmaceutical companies**
Hanati Tuoken	Boehringer Ingelheim, Ingelheim am Rhein, Germany	hanati.tuoken@boehringer-ingelheim.com	**Scientific Committee**	**Pharmaceutical companies**
Shameek Biswas	Bristol Myers Squibb, New York, NY, United States	Shameek.Biswas@bms.com	**Scientific Committee**	**Pharmaceutical companies**
Rion Pendergrass	Genentech, San Francisco, CA, United States	penders2@gene.com	**Scientific Committee**	**Pharmaceutical companies**
Margaret G. Ehm	GlaxoSmithKline, Collegeville, PA, United States	meg.g.ehm@gsk.com	**Scientific Committee**	**Pharmaceutical companies**
David Pulford	GlaxoSmithKline, Stevenage, United Kingdom	david.x.pulford@gsk.com	**Scientific Committee**	**Pharmaceutical companies**
Neha Raghavan	Merck, Kenilworth, NJ, United States	neha.raghavan@merck.com	**Scientific Committee**	**Pharmaceutical companies**
Adriana Huertas-Vazquez	Merck, Kenilworth, NJ, United States	adriana.huertas.vazquez@merck.com	**Scientific Committee**	**Pharmaceutical companies**
Jae-Hoon Sul	Merck, Kenilworth, NJ, United States	jae.hoon.sul@merck.com	**Scientific Committee**	**Pharmaceutical companies**
Anders Mälarstig	Pfizer, New York, NY, United States	anders.malarstig@pfizer.com	**Scientific Committee**	**Pharmaceutical companies**
Xinli Hu	Pfizer, New York, NY, United States	xinli.hu@pfizer.com	**Scientific Committee**	**Pharmaceutical companies**
Åsa Hedman	Pfizer, New York, NY, United States	asa.hedman@pfizer.com	**Scientific Committee**	**Pharmaceutical companies**
Katherine Klinger	Translational Sciences, Sanofi R&D, Framingham, MA, USA	katherine.klinger@sanofi.com	**Scientific Committee**	**Pharmaceutical companies**
Robert Graham	Maze Therapeutics, San Francisco, CA, United States	rgraham@mazetx.com	**Scientific Committee**	**Pharmaceutical companies**
Dawn Waterworth	Janssen Research & Development, LLC, Spring House, PA, United States	dwaterwo@its.jnj.com	**Scientific Committee**	**Pharmaceutical companies**
Nicole Renaud	Novartis Institutes for BioMedical Research, Cambridge, MA, United States	nicole.renaud@novartis.com	**Scientific Committee**	**Pharmaceutical companies**
Ma´en Obeidat	Novartis Institutes for BioMedical Research, Cambridge, MA, United States	maen.obeidat@novartis.com	**Scientific Committee**	**Pharmaceutical companies**
Jonathan Chung	Novartis Institutes for BioMedical Research, Cambridge, MA, United States	jonathan.chung@novartis.com	**Scientific Committee**	**Pharmaceutical companies**
Jonas Zierer	Novartis Institutes for BioMedical Research, Cambridge, MA, United States	jonas.zierer@novartis.com	**Scientific Committee**	**Pharmaceutical companies**
Mari Niemi	Novartis Institutes for BioMedical Research, Cambridge, MA, United States	mari.niemi@novartis.com	**Scientific Committee**	**Pharmaceutical companies**

Samuli Ripatti	Institute for Molecular Medicine Finland (FIMM), HiLIFE, University of Helsinki, Helsinki, Finland	samuli.ripatti@helsinki.fi	**Scientific Committee**	**University of Helsinki & Biobanks**
Johanna Schleutker	Auria Biobank / Univ. of Turku / Hospital District of Southwest Finland, Turku, Finland	johanna.schleutker@utu.fi	**Scientific Committee**	**University of Helsinki & Biobanks**
Markus Perola	THL Biobank / Finnish Institute for Health and Welfare (THL), Helsinki, Finland	markus.perola@thl.fi	**Scientific Committee**	**University of Helsinki & Biobanks**
Mikko Arvas	Finnish Red Cross Blood Service / Finnish Hematology Registry and Clinical Biobank, Helsinki, Finland	mikko.arvas@veripalvelu.fi	**Scientific Committee**	**University of Helsinki & Biobanks**
Olli Carpén	Helsinki Biobank / Helsinki University and Hospital District of Helsinki and Uusimaa, Helsinki	olli.carpen@helsinki.fi	**Scientific Committee**	**University of Helsinki & Biobanks**
Reetta Hinttala	Northern Finland Biobank Borealis / University of Oulu / Northern Ostrobothnia Hospital District, Oulu, Finland	reetta.hinttala@oulu.fi	**Scientific Committee**	**University of Helsinki & Biobanks**
Johannes Kettunen	Northern Finland Biobank Borealis / University of Oulu / Northern Ostrobothnia Hospital District, Oulu, Finland	johannes.kettunen@oulu.fi	**Scientific Committee**	**University of Helsinki & Biobanks**
Arto Mannermaa	Biobank of Eastern Finland / University of Eastern Finland / Northern Savo Hospital District, Kuopio, Finland	arto.mannermaa@uef.fi	**Scientific Committee**	**University of Helsinki & Biobanks**
Katriina Aalto-Setälä	Faculty of Medicine and Health Technology, Tampere University, Tampere, Finland	katriina.aalto-setala@tuni.fi	**Scientific Committee**	**University of Helsinki & Biobanks**
Mika Kähönen	Finnish Clinical Biobank Tampere / University of Tampere / Pirkanmaa Hospital District, Tampere, Finland	mika.kahonen@uta.fi	**Scientific Committee**	**University of Helsinki & Biobanks**
Jari Laukkanen	Central Finland Biobank / University of Jyväskylä / Central Finland Health Care District, Jyväskylä, Finland	jari.laukkanen@ksshp.fi	**Scientific Committee**	**University of Helsinki & Biobanks**
Johanna Mäkelä	FINBB - Finnish biobank cooperative	johanna.makela@finbb.fi	**Scientific Committee**	**University of Helsinki & Biobanks**
Reetta Kälviäinen	Northern Savo Hospital District, Kuopio, Finland	reetta.kalviainen@kuh.fi	**Clinical Groups**	**Neurology Group**
Valtteri Julkunen	Northern Savo Hospital District, Kuopio, Finland	valtteri.julkunen@kuh.fi	**Clinical Groups**	**Neurology Group**
Hilkka Soininen	Northern Savo Hospital District, Kuopio, Finland	hilkka.soininen@uef.fi	**Clinical Groups**	**Neurology Group**
Anne Remes	Northern Ostrobothnia Hospital District, Oulu, Finland	anne.remes@oulu.fi	**Clinical Groups**	**Neurology Group**
Mikko Hiltunen	University of Eastern Finland, Kuopio, Finland	mikko.hiltunen@uef.fi	**Clinical Groups**	**Neurology Group**
Jukka Peltola	Pirkanmaa Hospital District, Tampere, Finland	jukka.peltola@pshp.fi	**Clinical Groups**	**Neurology Group**
Minna Raivio	Hospital District of Helsinki and Uusimaa, Helsinki, Finland	minna.raivio@geri.fi	**Clinical Groups**	**Neurology Group**
Pentti Tienari	Hospital District of Helsinki and Uusimaa, Helsinki, Finland	pentti.tienari@hus.fi	**Clinical Groups**	**Neurology Group**
Juha Rinne	Hospital District of Southwest Finland, Turku, Finland	juha.rinne@tyks.fi	**Clinical Groups**	**Neurology Group**
Roosa Kallionpää	Hospital District of Southwest Finland, Turku, Finland	roosa.kallionpaa@tyks.fi	**Clinical Groups**	**Neurology Group**
Juulia Partanen	Institute for Molecular Medicine Finland, HiLIFE, University of Helsinki, Finland	juulia.partanen@helsinki.fi	**Clinical Groups**	**Neurology Group**
Adam Ziemann	Abbvie, Chicago, IL, United States	adam.ziemann@abbvie.com	**Clinical Groups**	**Neurology Group**
Nizar Smaoui	Abbvie, Chicago, IL, United States	nizar.smaoui@abbvie.com	**Clinical Groups**	**Neurology Group**
Anne Lehtonen	Abbvie, Chicago, IL, United States	anne.lehtonen@abbvie.com	**Clinical Groups**	**Neurology Group**
Susan Eaton	Biogen, Cambridge, MA, United States	susan.eaton@biogen.com	**Clinical Groups**	**Neurology Group**
Heiko Runz	Biogen, Cambridge, MA, United States	heiko.runz@biogen.com	**Clinical Groups**	**Neurology Group**
Sanni Lahdenperä	Biogen, Cambridge, MA, United States	sanni.lahdenpera@biogen.com	**Clinical Groups**	**Neurology Group**

Shameek Biswas	Bristol Myers Squibb, New York, NY, United States	shameek.biswas@bms.com	**Clinical Groups**	**Neurology Group**
Natalie Bowers	Genentech, San Francisco, CA, United States	bowersn1@gene.com	**Clinical Groups**	**Neurology Group**
Edmond Teng	Genentech, San Francisco, CA, United States	teng.edmond@gene.com	**Clinical Groups**	**Neurology Group**
Rion Pendergrass	Genentech, San Francisco, CA, United States	penders2@gene.com	**Clinical Groups**	**Neurology Group**
Fanli Xu	GlaxoSmithKline, Brentford, United Kingdom	chun-fang.2.xu@gsk.com	**Clinical Groups**	**Neurology Group**
David Pulford	GlaxoSmithKline, Stevenage, United Kingdom	david.x.pulford@gsk.com	**Clinical Groups**	**Neurology Group**
Kirsi Auro	GlaxoSmithKline, Espoo, Finland	kirsi.m.auro@gsk.com	**Clinical Groups**	**Neurology Group**
Laura Addis	GlaxoSmithKline, Brentford, United Kingdom	laura.x.addis@gsk.com	**Clinical Groups**	**Neurology Group**
John Eicher	GlaxoSmithKline, Brentford, United Kingdom	john.d.eicher@gsk.com	**Clinical Groups**	**Neurology Group**
Qingqin S Li	Janssen Research & Development, LLC, Titusville, NJ 08560, United States	QLi2@its.jnj.com	**Clinical Groups**	**Neurology Group**
Karen He	Janssen Research & Development, LLC, Spring House, PA, United States	khe2@its.jnj.com	**Clinical Groups**	**Neurology Group**
Ekaterina Khramtsova	Janssen Research & Development, LLC, Spring House, PA, United States	ekhramts@its.jnj.com	**Clinical Groups**	**Neurology Group**
Neha Raghavan	Merck, Kenilworth, NJ, United States	neha.raghavan@merck.com	**Clinical Groups**	**Neurology Group**
Martti Färkkilä	Hospital District of Helsinki and Uusimaa, Helsinki, Finland	martti.farkkila@hus.fi	**Clinical Groups**	**Gastroenterology Group**
Jukka Koskela	Hospital District of Helsinki and Uusimaa, Helsinki, Finland	jukka.koskela@helsinki.fi	**Clinical Groups**	**Gastroenterology Group**
Sampsa Pikkarainen	Hospital District of Helsinki and Uusimaa, Helsinki, Finland	sampsa.pikkarainen@hus.fi	**Clinical Groups**	**Gastroenterology Group**
Airi Jussila	Pirkanmaa Hospital District, Tampere, Finland	airi.jussila@pshp.fi	**Clinical Groups**	**Gastroenterology Group**
Katri Kaukinen	Pirkanmaa Hospital District, Tampere, Finland	katri.kaukinen@tuni.fi	**Clinical Groups**	**Gastroenterology Group**
Timo Blomster	Northern Ostrobothnia Hospital District, Oulu, Finland	timo.blomster@ppshp.fi	**Clinical Groups**	**Gastroenterology Group**
Mikko Kiviniemi	Northern Savo Hospital District, Kuopio, Finland	mikko.kiviniemi@kuh.fi	**Clinical Groups**	**Gastroenterology Group**
Markku Voutilainen	Hospital District of Southwest Finland, Turku, Finland	markku.voutilainen@tyks.fi	**Clinical Groups**	**Gastroenterology Group**
Mark Daly	Institute for Molecular Medicine, Finland (FIMM), HiLIFE, University of Helsinki, Helsinki, Finland; Broad Institute of MIT and Harvard; Massachusetts General Hospital	mark.daly@helsinki.fi	**Clinical Groups**	**Gastroenterology Group**
Jeffrey Waring	Abbvie, Chicago, IL, United States	jeff.waring@abbvie.com	**Clinical Groups**	**Gastroenterology Group**
Nizar Smaoui	Abbvie, Chicago, IL, United States	nizar.smaoui@abbvie.com	**Clinical Groups**	**Gastroenterology Group**
Fedik Rahimov	Abbvie, Chicago, IL, United States	fedik.rahimov@abbvie.com	**Clinical Groups**	**Gastroenterology Group**
Anne Lehtonen	Abbvie, Chicago, IL, United States	anne.lehtonen@abbvie.com	**Clinical Groups**	**Gastroenterology Group**
Tim Lu	Genentech, San Francisco, CA, United States	lut8@gene.com	**Clinical Groups**	**Gastroenterology Group**
Natalie Bowers	Genentech, San Francisco, CA, United States	bowersn1@gene.com	**Clinical Groups**	**Gastroenterology Group**
Rion Pendergrass	Genentech, San Francisco, CA, United States	penders2@gene.com	**Clinical Groups**	**Gastroenterology Group**
Linda McCarthy	GlaxoSmithKline, Brentford, United Kingdom	linda.c.mccarthy@gsk.com	**Clinical Groups**	**Gastroenterology Group**
Amy Hart	Janssen Research & Development, LLC, Spring House, PA, United States	ahart13@its.jnj.com	**Clinical Groups**	**Gastroenterology Group**
Meijian Guan	Janssen Research & Development, LLC, Spring House, PA, United States	mguan4@its.jnj.com	**Clinical Groups**	**Gastroenterology Group**
Jason Miller	Merck, Kenilworth, NJ, United States	jason.miller4@merck.com	**Clinical Groups**	**Gastroenterology Group**
Kirsi Kalpala	Pfizer, New York, NY, United States	kirsi.kalpala@pfizer.com	**Clinical Groups**	**Gastroenterology Group**

Melissa Miller	Pfizer, New York, NY, United States	melissa.r.miller@pfizer.com	**Clinical Groups**	**Gastroenterology Group**
Xinli Hu	Pfizer, New York, NY, United States	xinli.hu@pfizer.com	**Clinical Groups**	**Gastroenterology Group**
Kari Eklund	Hospital District of Helsinki and Uusimaa, Helsinki, Finland	kari.eklund@hus.fi	**Clinical Groups**	**Rheumatology Group**
Antti Palomäki	Hospital District of Southwest Finland, Turku, Finland	ajpalo@utu.fi	**Clinical Groups**	**Rheumatology Group**
Pia Isomäki	Pirkanmaa Hospital District, Tampere, Finland	pia.isomaki@pshp.fi	**Clinical Groups**	**Rheumatology Group**
Laura Pirilä	Hospital District of Southwest Finland, Turku, Finland	laura.pirila@fimnet.fi,laura.pirila@tyks.fi	**Clinical Groups**	**Rheumatology Group**
Oili Kaipiainen-Seppänen	Northern Savo Hospital District, Kuopio, Finland	oili.kaipiainen-seppanen@kuh.fi	**Clinical Groups**	**Rheumatology Group**
Johanna Huhtakangas	Northern Ostrobothnia Hospital District, Oulu, Finland	johanna.huhtakangas@kuh.fi	**Clinical Groups**	**Rheumatology Group**
Nina Mars	Institute for Molecular Medicine Finland (FIMM), HiLIFE, University of Helsinki, Helsinki, Finland	nina.mars@helsinki.fi	**Clinical Groups**	**Rheumatology Group**
Jeffrey Waring	Abbvie, Chicago, IL, United States	jeff.waring@abbvie.com	**Clinical Groups**	**Rheumatology Group**
Fedik Rahimov	Abbvie, Chicago, IL, United States	fedik.rahimov@abbvie.com	**Clinical Groups**	**Rheumatology Group**
Apinya Lertratanakul	Abbvie, Chicago, IL, United States	apinya.lertratanakul@abbvie.com	**Clinical Groups**	**Rheumatology Group**
Nizar Smaoui	Abbvie, Chicago, IL, United States	nizar.smaoui@abbvie.com	**Clinical Groups**	**Rheumatology Group**
Anne Lehtonen	Abbvie, Chicago, IL, United States	anne.lehtonen@abbvie.com	**Clinical Groups**	**Rheumatology Group**
Coralie Viollet	AstraZeneca, Cambridge, United Kingdom	coralie.viollet@astrazeneca.com	**Clinical Groups**	**Rheumatology Group**
Marla Hochfeld	Bristol Myers Squibb, New York, NY, United States	mhochfeld@celgene.com	**Clinical Groups**	**Rheumatology Group**
Natalie Bowers	Genentech, San Francisco, CA, United States	bowersn1@gene.com	**Clinical Groups**	**Rheumatology Group**
Rion Pendergrass	Genentech, San Francisco, CA, United States	penders2@gene.com	**Clinical Groups**	**Rheumatology Group**
Jorge Esparza Gordillo	GlaxoSmithKline, Brentford, United Kingdom	jorge.x.esparza-gordillo@gsk.com	**Clinical Groups**	**Rheumatology Group**
Kirsi Auro	GlaxoSmithKline, Espoo, Finland	kirsi.m.auro@gsk.com	**Clinical Groups**	**Rheumatology Group**
Dawn Waterworth	Janssen Research & Development, LLC, Spring House, PA, United States	dwaterwo@its.jnj.com	**Clinical Groups**	**Rheumatology Group**
Fabiana Farias	Merck, Kenilworth, NJ, United States	fabiana.farias@merck.com	**Clinical Groups**	**Rheumatology Group**
Kirsi Kalpala	Pfizer, New York, NY, United States	kirsi.kalpala@pfizer.com	**Clinical Groups**	**Rheumatology Group**
Nan Bing	Pfizer, New York, NY, United States	nan.bing@pfizer.com	**Clinical Groups**	**Rheumatology Group**
Xinli Hu	Pfizer, New York, NY, United States	xinli.hu@pfizer.com	**Clinical Groups**	**Rheumatology Group**
Tarja Laitinen	Pirkanmaa Hospital District, Tampere, Finland	tarja.laitinen@pshp.fi	**Clinical Groups**	**Pulmonology Group**
Margit Pelkonen	Northern Savo Hospital District, Kuopio, Finland	margit.pelkonen@kuh.fi	**Clinical Groups**	**Pulmonology Group**
Paula Kauppi	Hospital District of Helsinki and Uusimaa, Helsinki, Finland	paula.kauppi@hus.fi	**Clinical Groups**	**Pulmonology Group**
Hannu Kankaanranta	University of Gothenburg, Gothenburg, Sweden/ Seinäjoki Central Hospital, Seinäjoki, Finland/ Tampere University, Tampere, Finland	hannu.kankaanranta@tuni.fi	**Clinical Groups**	**Pulmonology Group**
Terttu Harju	Northern Ostrobothnia Hospital District, Oulu, Finland	terttu.harju@oulu.fi	**Clinical Groups**	**Pulmonology Group**
Riitta Lahesmaa	Hospital District of Southwest Finland, Turku, Finland	rilahes@utu.fi	**Clinical Groups**	**Pulmonology Group**

Nizar Smaoui	Abbvie, Chicago, IL, United States	nizar.smaoui@abbvie.com	**Clinical Groups**	**Pulmonology Group**
Coralie Viollet	AstraZeneca, Cambridge, United Kingdom	coralie.viollet@astrazeneca.com	**Clinical Groups**	**Pulmonology Group**
Susan Eaton	Biogen, Cambridge, MA, United States	susan.eaton@biogen.com	**Clinical Groups**	**Pulmonology Group**
Hubert Chen	Genentech, San Francisco, CA, United States	chenh37@gene.com	**Clinical Groups**	**Pulmonology Group**
Rion Pendergrass	Genentech, San Francisco, CA, United States	penders2@gene.com	**Clinical Groups**	**Pulmonology Group**
Natalie Bowers	Genentech, San Francisco, CA, United States	bowersn1@gene.com	**Clinical Groups**	**Pulmonology Group**
Joanna Betts	GlaxoSmithKline, Brentford, United Kingdom	joanna.c.betts@gsk.com	**Clinical Groups**	**Pulmonology Group**
Kirsi Auro	GlaxoSmithKline, Espoo, Finland	kirsi.m.auro@gsk.com	**Clinical Groups**	**Pulmonology Group**
Rajashree Mishra	GlaxoSmithKline, Brentford, United Kingdom	rajashree.x.mishra@gsk.com	**Clinical Groups**	**Pulmonology Group**
Majd Mouded	Novartis, Basel, Switzerland	majd.mouded@novartis.com	**Clinical Groups**	**Pulmonology Group**
Debby Ngo	Novartis, Basel, Switzerland	debby.ngo@novartis.com	**Clinical Groups**	**Pulmonology Group**
Teemu Niiranen	Finnish Institute for Health and Welfare (THL), Helsinki, Finland	teemu.niiranen@thl.fi	**Clinical Groups**	**Cardiometabolic Diseases Group**
Felix Vaura	Finnish Institute for Health and Welfare (THL), Helsinki, Finland	fechva@utu.fi	**Clinical Groups**	**Cardiometabolic Diseases Group**
Veikko Salomaa	Finnish Institute for Health and Welfare (THL), Helsinki, Finland	veikko.salomaa@thl.fi	**Clinical Groups**	**Cardiometabolic Diseases Group**
Kaj Metsärinne	Hospital District of Southwest Finland, Turku, Finland	kaj.metsarinne@tyks.fi	**Clinical Groups**	**Cardiometabolic Diseases Group**
Jenni Aittokallio	Hospital District of Southwest Finland, Turku, Finland	jemato@utu.fi	**Clinical Groups**	**Cardiometabolic Diseases Group**
Mika Kähönen	Pirkanmaa Hospital District, Tampere, Finland	mika.kahonen@uta.fi	**Clinical Groups**	**Cardiometabolic Diseases Group**
Jussi Hernesniemi	Pirkanmaa Hospital District, Tampere, Finland	jussi.hernesniemi@tuni.fi	**Clinical Groups**	**Cardiometabolic Diseases Group**
Daniel Gordin	Hospital District of Helsinki and Uusimaa, Helsinki, Finland	daniel.gordin@hus.fi	**Clinical Groups**	**Cardiometabolic Diseases Group**
Juha Sinisalo	Hospital District of Helsinki and Uusimaa, Helsinki, Finland	juha.sinisalo@hus.fi	**Clinical Groups**	**Cardiometabolic Diseases Group**
Marja-Riitta Taskinen	Hospital District of Helsinki and Uusimaa, Helsinki, Finland	marja-riitta.taskinen@helsinki.fi	**Clinical Groups**	**Cardiometabolic Diseases Group**
Tiinamaija Tuomi	Hospital District of Helsinki and Uusimaa, Helsinki, Finland	tiinamaija.tuomi@hus.fi	**Clinical Groups**	**Cardiometabolic Diseases Group**
Timo Hiltunen	Hospital District of Helsinki and Uusimaa, Helsinki, Finland	timo.hiltunen@hus.fi	**Clinical Groups**	**Cardiometabolic Diseases Group**
Jari Laukkanen	Central Finland Health Care District, Jyväskylä, Finland	jari.laukkanen@ksshp.fi	**Clinical Groups**	**Cardiometabolic Diseases Group**
Amanda Elliott	Institute for Molecular Medicine Finland (FIMM), HiLIFE, University of Helsinki, Helsinki, Finland; Broad Institute, Cambridge, MA, USA and Massachusetts General Hospital, Boston, MA, USA	aelliott@broadinstitute.org	**Clinical Groups**	**Cardiometabolic Diseases Group**
Mary Pat Reeve	Institute for Molecular Medicine Finland (FIMM), HiLIFE, University of Helsinki, Helsinki, Finland	mary.reeve@helsinki.fi	**Clinical Groups**	**Cardiometabolic Diseases Group**

Sanni Ruotsalainen	Institute for Molecular Medicine Finland (FIMM), HiLIFE, University of Helsinki, Helsinki, Finland	sanni.ruotsalainen@helsinki.fi	**Clinical Groups**	**Cardiometabolic Diseases Group**
Dirk Paul	Astra Zeneca, Cambridge, United Kingdom	dirk.paul@astrazeneca.com	**Clinical Groups**	**Cardiometabolic Diseases Group**
Natalie Bowers	Genentech, San Francisco, CA, United States	bowersn1@gene.com	**Clinical Groups**	**Cardiometabolic Diseases Group**
Rion Pendergrass	Genentech, San Francisco, CA, United States	penders2@gene.com	**Clinical Groups**	**Cardiometabolic Diseases Group**
Audrey Chu	GlaxoSmithKline, Brentford, United Kingdom	audrey.y.chu@gsk.com	**Clinical Groups**	**Cardiometabolic Diseases Group**
Kirsi Auro	GlaxoSmithKline, Espoo, Finland	kirsi.m.auro@gsk.com	**Clinical Groups**	**Cardiometabolic Diseases Group**
Dermot Reilly	Janssen Research & Development, LLC, Boston, MA, United States	dreill11@its.jnj.com	**Clinical Groups**	**Cardiometabolic Diseases Group**
Mike Mendelson	Novartis, Boston, MA, United States	mike.mendelson@novartis.com	**Clinical Groups**	**Cardiometabolic Diseases Group**
Jaakko Parkkinen	Pfizer, New York, NY, United States	jaakko.parkkinen@pfizer.com	**Clinical Groups**	**Cardiometabolic Diseases Group**
Melissa Miller	Pfizer, New York, NY, United States	melissa.r.miller@pfizer.com	**Clinical Groups**	**Cardiometabolic Diseases Group**
Tuomo Meretoja	Department of Breast Surgery, Helsinki University Hospital Comprehensive Cancer Center and University of Helsinki, Helsinki, Finland	tuomo.meretoja@hus.fi	**Clinical Groups**	**Oncology Group**
Heikki Joensuu	Department of Oncology, Helsinki University Hospital Comprehensive Cancer Center and University of Helsinki, Helsinki, Finland	heikki.joensuu@hus.fi	**Clinical Groups**	**Oncology Group**
Olli Carpén	Hospital District of Helsinki and Uusimaa, Helsinki, Finland	olli.carpen@helsinki.fi	**Clinical Groups**	**Oncology Group**
Johanna Mattson	Hospital District of Helsinki and Uusimaa, Helsinki, Finland	johanna.mattson@hus.fi	**Clinical Groups**	**Oncology Group**
Eveliina Salminen	Hospital District of Helsinki and Uusimaa, Helsinki, Finland	eveliina.e.salminen@hus.fi	**Clinical Groups**	**Oncology Group**
Annika Auranen	Pirkanmaa Hospital District , Tampere, Finland	anaura@utu.fi	**Clinical Groups**	**Oncology Group**
Peeter Karihtala	Department of Oncology, Helsinki University Hospital Comprehensive Cancer Center and University of Helsinki, Helsinki, Finland	peeter.karihtala@hus.fi	**Clinical Groups**	**Oncology Group**
Päivi Auvinen	Northern Savo Hospital District, Kuopio, Finland	paivi.auvinen@kuh.fi	**Clinical Groups**	**Oncology Group**
Klaus Elenius	Hospital District of Southwest Finland, Turku, Finland	klaus.elenius@utu.fi	**Clinical Groups**	**Oncology Group**
Johanna Schleutker	Hospital District of Southwest Finland, Turku, Finland	johanna.schleutker@utu.fi	**Clinical Groups**	**Oncology Group**
Esa Pitkänen	Institute for Molecular Medicine Finland (FIMM), HiLIFE, University of Helsinki, Helsinki, Finland	esa.pitkanen@helsinki.fi	**Clinical Groups**	**Oncology Group**
Nina Mars	Institute for Molecular Medicine Finland (FIMM), HiLIFE, University of Helsinki, Helsinki, Finland	nina.mars@helsinki.fi	**Clinical Groups**	**Oncology Group**
Mark Daly	Institute for Molecular Medicine Finland (FIMM), HiLIFE, University of Helsinki, Helsinki, Finland; Broad Institute of MIT and Harvard; Massachusetts General Hospital	mark.daly@helsinki.fi	**Clinical Groups**	**Oncology Group**
Relja Popovic	Abbvie, Chicago, IL, United States	relja.popovic@abbvie.com	**Clinical Groups**	**Oncology Group**
Jeffrey Waring	Abbvie, Chicago, IL, United States	jeff.waring@abbvie.com	**Clinical Groups**	**Oncology Group**
Bridget Riley-Gillis	Abbvie, Chicago, IL, United States	bridget.rileygillis@abbvie.com	**Clinical Groups**	**Oncology Group**
Anne Lehtonen	Abbvie, Chicago, IL, United States	anne.lehtonen@abbvie.com	**Clinical Groups**	**Oncology Group**
Margarete Fabre	AstraZeneca, Cambridge, United Kingdom	margarete.fabre@astrazeneca.com	**Clinical Groups**	**Oncology Group**

Jennifer Schutzman	Genentech, San Francisco, CA, United States	schutzman.jennifer@gene.com	**Clinical Groups**	**Oncology Group**
Natalie Bowers	Genentech, San Francisco, CA, United States	bowersn1@gene.com	**Clinical Groups**	**Oncology Group**
Rion Pendergrass	Genentech, San Francisco, CA, United States	penders2@gene.com	**Clinical Groups**	**Oncology Group**
Diptee Kulkarni	GlaxoSmithKline, Brentford, United Kingdom	diptee.a.kulkarni@gsk.com	**Clinical Groups**	**Oncology Group**
Kirsi Auro	GlaxoSmithKline, Espoo, Finland	kirsi.m.auro@gsk.com	**Clinical Groups**	**Oncology Group**
Alessandro Porello	Janssen Research & Development, LLC, Spring House, PA, United States	APorrell@ITS.JNJ.com	**Clinical Groups**	**Oncology Group**
Andrey Loboda	Merck, Kenilworth, NJ, United States	andrey_loboda@merck.com	**Clinical Groups**	**Oncology Group**
Heli Lehtonen	Pfizer, New York, NY, United States	heli.lehtonen@pfizer.com	**Clinical Groups**	**Oncology Group**
Stefan McDonough	Pfizer, New York, NY, United States	stefan.McDonough@pfizer.com	**Clinical Groups**	**Oncology Group**
Sauli Vuoti	Janssen-Cilag Oy, Espoo, Finland	svuoti@its.jnj.com	**Clinical Groups**	**Oncology Group**
Kai Kaarniranta	Northern Savo Hospital District, Kuopio, Finland; Department of Molecular Genetics, University of Lodz, Lodz, Poland	kai.kaarniranta@uef.fi	**Clinical Groups**	**Opthalmology Group**
Joni A Turunen	Helsinki University Hospital and University of Helsinki, Helsinki, Finland; Eye Genetics Group, Folkhälsan Research Center, Helsinki, Finland	joni.turunen@helsinki.fi	**Clinical Groups**	**Opthalmology Group**
Terhi Ollila	Hospital District of Helsinki and Uusimaa, Helsinki, Finland	terhi.ollila@hus.fi	**Clinical Groups**	**Opthalmology Group**
Hannu Uusitalo	Pirkanmaa Hospital District, Tampere, Finland	hannu.uusitalo@tuni.fi	**Clinical Groups**	**Opthalmology Group**
Juha Karjalainen	Institute for Molecular Medicine Finland (FIMM), HiLIFE, University of Helsinki, Helsinki, Finland	juha.karjalainen@helsinki.fi	**Clinical Groups**	**Opthalmology Group**
Esa Pitkänen	Institute for Molecular Medicine Finland (FIMM), HiLIFE, University of Helsinki, Helsinki, Finland	esa.pitkanen@helsinki.fi	**Clinical Groups**	**Opthalmology Group**
Mengzhen Liu	Abbvie, Chicago, IL, United States	mengzhen.liu@abbvie.com	**Clinical Groups**	**Opthalmology Group**
Heiko Runz	Biogen, Cambridge, MA, United States	heiko.runz@biogen.com	**Clinical Groups**	**Opthalmology Group**
Stephanie Loomis	Biogen, Cambridge, MA, United States	stephanie.loomis@biogen.com	**Clinical Groups**	**Opthalmology Group**
Erich Strauss	Genentech, San Francisco, CA, United States	strauss.erich@gene.com	**Clinical Groups**	**Opthalmology Group**
Natalie Bowers	Genentech, San Francisco, CA, United States	bowersn1@gene.com	**Clinical Groups**	**Opthalmology Group**
Hao Chen	Genentech, San Francisco, CA, United States	haoc@gene.com	**Clinical Groups**	**Opthalmology Group**
Rion Pendergrass	Genentech, San Francisco, CA, United States	penders2@gene.com	**Clinical Groups**	**Opthalmology Group**
Kaisa Tasanen	Northern Ostrobothnia Hospital District, Oulu, Finland	kaisa.tasanen-maatta@oulu.fi	**Clinical Groups**	**Dermatology Group**
Laura Huilaja	Northern Ostrobothnia Hospital District, Oulu, Finland	laura.huilaja@oulu.fi	**Clinical Groups**	**Dermatology Group**
Katariina Hannula-Jouppi	Hospital District of Helsinki and Uusimaa, Helsinki, Finland	katariina.hannula-jouppi@hus.fi	**Clinical Groups**	**Dermatology Group**
Teea Salmi	Pirkanmaa Hospital District, Tampere, Finland	teea.salmi@pshp.fi	**Clinical Groups**	**Dermatology Group**
Sirkku Peltonen	Hospital District of Southwest Finland, Turku, Finland	sipelto@utu.fi	**Clinical Groups**	**Dermatology Group**
Leena Koulu	Hospital District of Southwest Finland, Turku, Finland	leena.koulu@tyks.fi	**Clinical Groups**	**Dermatology Group**
Nizar Smaoui	Abbvie, Chicago, IL, United States	nizar.smaoui@abbvie.com	**Clinical Groups**	**Dermatology Group**
Fedik Rahimov	Abbvie, Chicago, IL, United States	fedik.rahimov@abbvie.com	**Clinical Groups**	**Dermatology Group**

Anne Lehtonen	Abbvie, Chicago, IL, United States	anne.lehtonen@abbvie.com	**Clinical Groups**	**Dermatology Group**
David Choy	Genentech, San Francisco, CA, United States	choy.david@gene.com	**Clinical Groups**	**Dermatology Group**
Rion Pendergrass	Genentech, San Francisco, CA, United States	penders2@gene.com	**Clinical Groups**	**Dermatology Group**
Dawn Waterworth	Janssen Research & Development, LLC, Spring House, PA, United States	dwaterwo@its.jnj.com	**Clinical Groups**	**Dermatology Group**
Kirsi Kalpala	Pfizer, New York, NY, United States	kirsi.kalpala@pfizer.com	**Clinical Groups**	**Dermatology Group**
Ying Wu	Pfizer, New York, NY, United States	ying.wu3@pfizer.com	**Clinical Groups**	**Dermatology Group**
Pirkko Pussinen	Hospital District of Helsinki and Uusimaa, Helsinki, Finland	pirkko.pussinen@helsinki.fi	**Clinical Groups**	**Odontology Group**
Aino Salminen	Hospital District of Helsinki and Uusimaa, Helsinki, Finland	aino.m.salminen@helsinki.fi	**Clinical Groups**	**Odontology Group**
Tuula Salo	Hospital District of Helsinki and Uusimaa, Helsinki, Finland	tuula.salo@helsinki.fi	**Clinical Groups**	**Odontology Group**
David Rice	Hospital District of Helsinki and Uusimaa, Helsinki, Finland	david.rice@helsinki.fi	**Clinical Groups**	**Odontology Group**
Pekka Nieminen	Hospital District of Helsinki and Uusimaa, Helsinki, Finland	pekka.nieminen@helsinki.fi	**Clinical Groups**	**Odontology Group**
Ulla Palotie	Hospital District of Helsinki and Uusimaa, Helsinki, Finland	ulla.palotie@helsinki.fi	**Clinical Groups**	**Odontology Group**
Maria Siponen	Northern Savo Hospital District, Kuopio, Finland	maria.siponen@uef.fi	**Clinical Groups**	**Odontology Group**
Liisa Suominen	Northern Savo Hospital District, Kuopio, Finland	liisa.suominen@uef.fi	**Clinical Groups**	**Odontology Group**
Päivi Mäntylä	Northern Savo Hospital District, Kuopio, Finland	paivi.mantyla@uef.fi	**Clinical Groups**	**Odontology Group**
Ulvi Gursoy	Hospital District of Southwest Finland, Turku, Finland	ulvi.gursoy@utu.fi	**Clinical Groups**	**Odontology Group**
Vuokko Anttonen	Northern Ostrobothnia Hospital District, Oulu, Finland	vuokko.anttonen@oulu.fi	**Clinical Groups**	**Odontology Group**
Kirsi Sipilä	Research Unit of Oral Health Sciences Faculty of Medicine, University of Oulu, Oulu, Finland; Medical Research Center, Oulu, Oulu University Hospital and University of Oulu, Oulu, Finland	kirsi.sipila@oulu.fi	**Clinical Groups**	**Odontology Group**
Rion Pendergrass	Genentech, San Francisco, CA, United States	pendergrass.sarah@gene.com	**Clinical Groups**	**Odontology Group**
Hannele Laivuori	Institute for Molecular Medicine Finland (FIMM), HiLIFE, University of Helsinki, Helsinki, Finland	hannele.laivuori@helsinki.fi	**Clinical Groups**	**Women's Health and Reproduction Group**
Venla Kurra	Pirkanmaa Hospital District, Tampere, Finland	venla.kurra@tuni.fi	**Clinical Groups**	**Women's Health and Reproduction Group**
Laura Kotaniemi-Talonen	Pirkanmaa Hospital District, Tampere, Finland	laura.kotaniemi-talonen@tuni.fi	**Clinical Groups**	**Women's Health and Reproduction Group**
Oskari Heikinheimo	Hospital District of Helsinki and Uusimaa, Helsinki, Finland	oskari.heikinheimo@helsinki.fi	**Clinical Groups**	**Women's Health and Reproduction Group**
Ilkka Kalliala	Hospital District of Helsinki and Uusimaa, Helsinki, Finland	ilkka.kalliala@hus.fi	**Clinical Groups**	**Women's Health and Reproduction Group**
Lauri Aaltonen	Hospital District of Helsinki and Uusimaa, Helsinki, Finland	lauri.aaltonen@helsinki.fi	**Clinical Groups**	**Women's Health and Reproduction Group**
Varpu Jokimaa	Hospital District of Southwest Finland, Turku, Finland	varpu.jokimaa@utu.fi	**Clinical Groups**	**Women's Health and Reproduction Group**
Johannes Kettunen	Northern Ostrobothnia Hospital District, Oulu, Finland	Johannes.Kettunen@oulu.fi	**Clinical Groups**	**Women's Health and**

				**Reproduction Group**
Marja Vääräsmäki	Northern Ostrobothnia Hospital District, Oulu, Finland	marja.vaarasmaki@oulu.fi	**Clinical Groups**	**Women's Health and Reproduction Group**
Outi Uimari	Northern Ostrobothnia Hospital District, Oulu, Finland	outi.uimari@oulu.fi	**Clinical Groups**	**Women's Health and Reproduction Group**
Laure Morin-Papunen	Northern Ostrobothnia Hospital District, Oulu, Finland	lmp@cc.oulu.fi	**Clinical Groups**	**Women's Health and Reproduction Group**
Maarit Niinimäki	Northern Ostrobothnia Hospital District, Oulu, Finland	maarit.niinimaki@oulu.fi	**Clinical Groups**	**Women's Health and Reproduction Group**
Terhi Piltonen	Northern Ostrobothnia Hospital District, Oulu, Finland	terhi.piltonen@oulu.fi	**Clinical Groups**	**Women's Health and Reproduction Group**
Katja Kivinen	Institute for Molecular Medicine Finland (FIMM), HiLIFE, University of Helsinki, Helsinki, Finland	katja.kivinen@helsinki.fi	**Clinical Groups**	**Women's Health and Reproduction Group**
Elisabeth Widen	Institute for Molecular Medicine Finland (FIMM), HiLIFE, University of Helsinki, Helsinki, Finland	elisabeth.widen@helsinki.fi	**Clinical Groups**	**Women's Health and Reproduction Group**
Taru Tukiainen	Institute for Molecular Medicine Finland (FIMM), HiLIFE, University of Helsinki, Helsinki, Finland	taru.tukiainen@helsinki.fi	**Clinical Groups**	**Women's Health and Reproduction Group**
Mary Pat Reeve	Institute for Molecular Medicine Finland (FIMM), HiLIFE, University of Helsinki, Helsinki, Finland	mary.reeve@helsinki.fi	**Clinical Groups**	**Women's Health and Reproduction Group**
Mark Daly	Institute for Molecular Medicine Finland (FIMM), HiLIFE, University of Helsinki, Helsinki, Finland; Broad Institute of MIT and Harvard; Massachusetts General Hospital	mark.daly@helsinki.fi	**Clinical Groups**	**Women's Health and Reproduction Group**
Niko Välimäki	University of Helsinki, Helsinki, Finland	niko.valimaki@helsinki.fi	**Clinical Groups**	**Women's Health and Reproduction Group**
Eija Laakkonen	University of Jyväskylä, Jyväskylä, Finland	eija.k.laakkonen@jyu.fi	**Clinical Groups**	**Women's Health and Reproduction Group**
Jaakko Tyrmi	University of Oulu, Oulu, Finland / University of Tampere, Tampere, Finland	jaakko.tyrmi@oulu.fi	**Clinical Groups**	**Women's Health and Reproduction Group**
Heidi Silven	University of Oulu, Oulu, Finland	heidi.silven@student.oulu.fi	**Clinical Groups**	**Women's Health and Reproduction Group**
Eeva Sliz	University of Oulu, Oulu, Finland	eeva.sliz@oulu.fi	**Clinical Groups**	**Women's Health and Reproduction Group**
Riikka Arffman	University of Oulu, Oulu, Finland	riikka.arffman@oulu.fi	**Clinical Groups**	**Women's Health and Reproduction Group**
Susanna Savukoski	University of Oulu, Oulu, Finland	susanna.savukoski@oulu.fi	**Clinical Groups**	**Women's Health and Reproduction Group**
Triin Laisk	Estonian biobank, Tartu, Estonia	triin.laisk@ut.ee	**Clinical Groups**	**Women's Health and**

				**Reproduction Group**
Natalia Pujol	Estonian biobank, Tartu, Estonia	natalia.pujolgualdo@oulu.fi	**Clinical Groups**	**Women's Health and Reproduction Group**
Mengzhen Liu	Abbvie, Chicago, IL, United States	mengzhen.liu@abbvie.com	**Clinical Groups**	**Women's Health and Reproduction Group**
Bridget Riley-Gillis	Abbvie, Chicago, IL, United States	bridget.rileygillis@abbvie.com	**Clinical Groups**	**Women's Health and Reproduction Group**
Rion Pendergrass	Genentech, San Francisco, CA, United States	penders2@gene.com	**Clinical Groups**	**Women's Health and Reproduction Group**
Janet Kumar	GlaxoSmithKline, Collegeville, PA, United States	janet.x.kumar@gsk.com	**Clinical Groups**	**Women's Health and Reproduction Group**
Kirsi Auro	GlaxoSmithKline, Espoo, Finland	kirsi.m.auro@gsk.com	**Clinical Groups**	**Women's Health and Reproduction Group**
Iiris Hovatta	University of Helsinki, Finland	iiris.hovatta@helsinki.fi	**Clinical Groups**	**Depression group**
Chia-Yen Chen	Biogen, Cambridge, MA, United States	chiayen.chen@biogen.com	**Clinical Groups**	**Depression group**
Erkki Isometsä	Hospital District of Helsinki and Uusimaa, Helsinki, Finland	erkki.isometsa@hus.fi	**Clinical Groups**	**Depression group**
Hanna Ollila	Institute for Molecular Medicine Finland (FIMM), HiLIFE, University of Helsinki, Helsinki, Finland	hanna.m.ollila@helsinki.fi	**Clinical Groups**	**Depression group**
Jaana Suvisaari	Finnish Institute for Health and Welfare (THL), Helsinki, Finland	jaana.suvisaari@thl.fi	**Clinical Groups**	**Depression group**
Antti Mäkitie	Department of Otorhinolaryngology - Head and Neck Surgery, University of Helsinki and Helsinki University Hospital, Helsinki, Finland	antti.makitie@helsinki.fi	**Clinical Groups**	**ENT (ear, nose and throath) Group**
Argyro Bizaki-Vallaskangas	Pirkanmaa Hospital District, Tampere, Finland	argyro.bizaki-vallaskangas@tuni.fi	**Clinical Groups**	**ENT (ear, nose and throath) Group**
Sanna Toppila-Salmi	University of Eastern Finland and Kuopio University Hospital, Department of Otorhinolaryngology, Kuopio, Finland and Department of Allergy, Helsinki University Hospital and University of Helsinki, Finland	sanna.salmi@helsinki.fi	**Clinical Groups**	**ENT (ear, nose and throath) Group**
Tytti Willberg	Hospital District of Southwest Finland, Turku, Finland	tytti.willberg@tyks.fi	**Clinical Groups**	**ENT (ear, nose and throath) Group**
Elmo Saarentaus	Institute for Molecular Medicine Finland (FIMM), HiLIFE, University of Helsinki, Helsinki, Finland	elmo.saarentaus@helsinki.fi	**Clinical Groups**	**ENT (ear, nose and throath) Group**
Antti Aarnisalo	Hospital District of Helsinki and Uusimaa, Helsinki, Finland	antti.aarnisalo@hus.fi	**Clinical Groups**	**ENT (ear, nose and throath) Group**
Eveliina Salminen	Hospital District of Helsinki and Uusimaa, Helsinki, Finland	eveliina.e.salminen@hus.fi	**Clinical Groups**	**ENT (ear, nose and throath) Group**
Elisa Rahikkala	Northern Ostrobothnia Hospital District, Oulu, Finland	elisa.rahikkala@ppshp.fi	**Clinical Groups**	**ENT (ear, nose and throath) Group**
Johannes Kettunen	Northern Ostrobothnia Hospital District, Oulu, Finland	johannes.kettunen@oulu.fi	**Clinical Groups**	**ENT (ear, nose and throath) Group**

Kristiina Aittomäki	Department of Medical Genetics, Helsinki University Central Hospital, Helsinki, Finland	kristiina.aittomaki@helsinki.fi	**Clinical Groups**	**POI (premature ovarian failure) Group**
Fredrik Åberg	Transplantation and Liver Surgery Clinic, Helsinki University Hospital, Helsinki University, Helsinki, Finland	fredrik.aberg@helsinki.fi	**Clinical Groups**	**LiverScore Group**
Mitja Kurki	Institute for Molecular Medicine Finland (FIMM), HiLIFE, University of Helsinki, Helsinki, Finland; Broad Institute, Cambridge, MA, United States	mkurki@broadinstitute.org	**FinnGen Analysis working group**	**FinnGen Analysis working group**
Samuli Ripatti	Institute for Molecular Medicine Finland (FIMM), HiLIFE, University of Helsinki, Helsinki, Finland	samuli.ripatti@helsinki.fi	**FinnGen Analysis working group**	**FinnGen Analysis working group**
Mark Daly	Institute for Molecular Medicine, Finland (FIMM), HiLIFE, University of Helsinki, Helsinki, Finland; Broad Institute of MIT and Harvard; Massachusetts General Hospital	mark.daly@helsinki.fi	**FinnGen Analysis working group**	**FinnGen Analysis working group**
Juha Karjalainen	Institute for Molecular Medicine Finland (FIMM), HiLIFE, University of Helsinki, Helsinki, Finland	juha.karjalainen@helsinki.fi	**FinnGen Analysis working group**	**FinnGen Analysis working group**
Aki Havulinna	Institute for Molecular Medicine Finland (FIMM), HiLIFE, University of Helsinki, Helsinki, Finland; Finnish Institute for Health and Welfare (THL), Helsinki, Finland	aki.havulinna@helsinki.fi	**FinnGen Analysis working group**	**FinnGen Analysis working group**
Juha Mehtonen	Institute for Molecular Medicine Finland (FIMM), HiLIFE, University of Helsinki, Helsinki, Finland	juha.mehtonen@helsinki.fi	**FinnGen Analysis working group**	**FinnGen Analysis working group**
Priit Palta	Institute for Molecular Medicine Finland (FIMM), HiLIFE, University of Helsinki, Helsinki, Finland	priit.palta@helsinki.fi	**FinnGen Analysis working group**	**FinnGen Analysis working group**
Shabbeer Hassan	Institute for Molecular Medicine Finland (FIMM), HiLIFE, University of Helsinki, Helsinki, Finland	shabbeer.hassan@helsinki.fi	**FinnGen Analysis working group**	**FinnGen Analysis working group**
Pietro Della Briotta Parolo	Institute for Molecular Medicine Finland (FIMM), HiLIFE, University of Helsinki, Helsinki, Finland	pietro.dellabriottaparolo@helsinki.fi	**FinnGen Analysis working group**	**FinnGen Analysis working group**
Wei Zhou	Broad Institute, Cambridge, MA, United States	wzhou@broadinstitute.org	**FinnGen Analysis working group**	**FinnGen Analysis working group**
Mutaamba Maasha	Broad Institute, Cambridge, MA, United States	mmaasha@broadinstitute.org	**FinnGen Analysis working group**	**FinnGen Analysis working group**
Shabbeer Hassan	Institute for Molecular Medicine Finland (FIMM), HiLIFE, University of Helsinki, Helsinki, Finland	shabbeer.hassan@helsinki.fi	**FinnGen Analysis working group**	**FinnGen Analysis working group**
Susanna Lemmelä	Institute for Molecular Medicine Finland (FIMM), HiLIFE, University of Helsinki, Helsinki, Finland	susanna.lemmela@helsinki.fi	**FinnGen Analysis working group**	**FinnGen Analysis working group**
Manuel Rivas	University of Stanford, Stanford, CA, United States	mrivas@stanford.edu	**FinnGen Analysis working group**	**FinnGen Analysis working group**
Aarno Palotie	Institute for Molecular Medicine Finland (FIMM), HiLIFE, University of Helsinki, Helsinki, Finland	aarno.palotie@helsinki.fi	**FinnGen Analysis working group**	**FinnGen Analysis working group**
Aoxing Liu	Institute for Molecular Medicine Finland (FIMM), HiLIFE, University of Helsinki, Helsinki, Finland	aoxing.liu@helsinki.fi	**FinnGen Analysis working group**	**FinnGen Analysis working group**

Arto Lehisto	Institute for Molecular Medicine Finland (FIMM), HiLIFE, University of Helsinki, Helsinki, Finland	arto.lehisto@helsinki.fi	**FinnGen Analysis working group**	**FinnGen Analysis working group**
Andrea Ganna	Institute for Molecular Medicine Finland (FIMM), HiLIFE, University of Helsinki, Helsinki, Finland	aganna@broadinstitute.org	**FinnGen Analysis working group**	**FinnGen Analysis working group**
Vincent Llorens	Institute for Molecular Medicine Finland (FIMM), HiLIFE, University of Helsinki, Helsinki, Finland	vincent.llorens@helsinki.fi	**FinnGen Analysis working group**	**FinnGen Analysis working group**
Hannele Laivuori	Institute for Molecular Medicine Finland (FIMM), HiLIFE, University of Helsinki, Helsinki, Finland	hannele.laivuori@helsinki.fi	**FinnGen Analysis working group**	**FinnGen Analysis working group**
Taru Tukiainen	Institute for Molecular Medicine Finland (FIMM), HiLIFE, University of Helsinki, Helsinki, Finland	taru.tukiainen@helsinki.fi	**FinnGen Analysis working group**	**FinnGen Analysis working group**
Mary Pat Reeve	Institute for Molecular Medicine Finland (FIMM), HiLIFE, University of Helsinki, Helsinki, Finland	mary.reeve@helsinki.fi	**FinnGen Analysis working group**	**FinnGen Analysis working group**
Henrike Heyne	Institute for Molecular Medicine Finland (FIMM), HiLIFE, University of Helsinki, Helsinki, Finland	hheyne@broadinstitute.org	**FinnGen Analysis working group**	**FinnGen Analysis working group**
Nina Mars	Institute for Molecular Medicine Finland (FIMM), HiLIFE, University of Helsinki, Helsinki, Finland	nina.mars@helsinki.fi	**FinnGen Analysis working group**	**FinnGen Analysis working group**
Joel Rämö	Institute for Molecular Medicine Finland (FIMM), HiLIFE, University of Helsinki, Helsinki, Finland	joel.ramo@helsinki.fi	**FinnGen Analysis working group**	**FinnGen Analysis working group**
Elmo Saarentaus	Institute for Molecular Medicine Finland (FIMM), HiLIFE, University of Helsinki, Helsinki, Finland	elmo.saarentaus@helsinki.fi	**FinnGen Analysis working group**	**FinnGen Analysis working group**
Hanna Ollila	Institute for Molecular Medicine Finland (FIMM), HiLIFE, University of Helsinki, Helsinki, Finland	hanna.m.ollila@helsinki.fi	**FinnGen Analysis working group**	**FinnGen Analysis working group**
Rodos Rodosthenous	Institute for Molecular Medicine Finland (FIMM), HiLIFE, University of Helsinki, Helsinki, Finland	rodos.rodosthenous@helsinki.fi	**FinnGen Analysis working group**	**FinnGen Analysis working group**
Satu Strausz	Institute for Molecular Medicine Finland (FIMM), HiLIFE, University of Helsinki, Helsinki, Finland	satu.strausz@helsinki.fi	**FinnGen Analysis working group**	**FinnGen Analysis working group**
Tuula Palotie	University of Helsinki and Hospital District of Helsinki and Uusimaa, Helsinki, Finland	tuula.palotie@helsinki.fi	**FinnGen Analysis working group**	**FinnGen Analysis working group**
Kimmo Palin	University of Helsinki, Helsinki, Finland	kimmo.palin@helsinki.fi	**FinnGen Analysis working group**	**FinnGen Analysis working group**
Javier Garcia-Tabuenca	University of Tampere, Tampere, Finland	javier.graciatabuenca@tuni.fi	**FinnGen Analysis working group**	**FinnGen Analysis working group**
Harri Siirtola	University of Tampere, Tampere, Finland	harri.siirtola@tuni.fi	**FinnGen Analysis working group**	**FinnGen Analysis working group**
Tuomo Kiiskinen	Institute for Molecular Medicine Finland (FIMM), HiLIFE, University of Helsinki, Helsinki, Finland	tuomo.kiiskinen@helsinki.fi	**FinnGen Analysis working group**	**FinnGen Analysis working group**

Jiwoo Lee	Institute for Molecular Medicine Finland (FIMM), HiLIFE, University of Helsinki, Helsinki, Finland; Broad Institute, Cambridge, MA, United States	jiwoo.lee@helsinki.fi	**FinnGen Analysis working group**	**FinnGen Analysis working group**
Kristin Tsuo	Institute for Molecular Medicine Finland (FIMM), HiLIFE, University of Helsinki, Helsinki, Finland; Broad Institute, Cambridge, MA, United States	kristintsuo@fas.harvard.edu	**FinnGen Analysis working group**	**FinnGen Analysis working group**
Amanda Elliott	Institute for Molecular Medicine Finland (FIMM), HiLIFE, University of Helsinki, Helsinki, Finland; Broad Institute, Cambridge, MA, USA and Massachusetts General Hospital, Boston, MA, USA	aelliott@broadinstitute.org	**FinnGen Analysis working group**	**FinnGen Analysis working group**
Kati Kristiansson	THL Biobank / Finnish Institute for Health and Welfare (THL), Helsinki, Finland	kati.kristiansson@thl.fi	**FinnGen Analysis working group**	**FinnGen Analysis working group**
Mikko Arvas	Finnish Red Cross Blood Service / Finnish Hematology Registry and Clinical Biobank, Helsinki, Finland	mikko.arvas@veripalvelu.fi	**FinnGen Analysis working group**	**FinnGen Analysis working group**
Kati Hyvärinen	Finnish Red Cross Blood Service, Helsinki, Finland	kati.hyvarinen@veripalvelu.fi	**FinnGen Analysis working group**	**FinnGen Analysis working group**
Jarmo Ritari	Finnish Red Cross Blood Service, Helsinki, Finland	jarmo.ritari@veripalvelu.fi	**FinnGen Analysis working group**	**FinnGen Analysis working group**
Olli Carpén	Helsinki Biobank / Helsinki University and Hospital District of Helsinki and Uusimaa, Helsinki	olli.carpen@helsinki.fi	**FinnGen Analysis working group**	**FinnGen Analysis working group**
Johannes Kettunen	Northern Finland Biobank Borealis / University of Oulu / Northern Ostrobothnia Hospital District, Oulu, Finland	johannes.kettunen@oulu.fi	**FinnGen Analysis working group**	**FinnGen Analysis working group**
Katri Pylkäs	University of Oulu, Oulu, Finland	katri.pylkas@oulu.fi	**FinnGen Analysis working group**	**FinnGen Analysis working group**
Eeva Sliz	University of Oulu, Oulu, Finland	eeva.sliz@oulu.fi	**FinnGen Analysis working group**	**FinnGen Analysis working group**
Minna Karjalainen	University of Oulu, Oulu, Finland	minna.k.karjalainen@oulu.fi	**FinnGen Analysis working group**	**FinnGen Analysis working group**
Tuomo Mantere	Northern Finland Biobank Borealis / University of Oulu / Northern Ostrobothnia Hospital District, Oulu, Finland	tuomo.mantere@oulu.fi	**FinnGen Analysis working group**	**FinnGen Analysis working group**
Eeva Kangasniemi	Finnish Clinical Biobank Tampere / University of Tampere / Pirkanmaa Hospital District, Tampere, Finland	eeva.kangasniemi@pshp.fi	**FinnGen Analysis working group**	**FinnGen Analysis working group**
Sami Heikkinen	University of Eastern Finland, Kuopio, Finland	sami.heikkinen@uef.fi	**FinnGen Analysis working group**	**FinnGen Analysis working group**
Arto Mannermaa	Biobank of Eastern Finland / University of Eastern Finland / Northern Savo Hospital District, Kuopio, Finland	arto.mannermaa@uef.fi	**FinnGen Analysis working group**	**FinnGen Analysis working group**
Eija Laakkonen	University of Jyväskylä, Jyväskylä, Finland	eija.k.laakkonen@jyu.fi	**FinnGen Analysis working group**	**FinnGen Analysis working group**
Nina Pitkänen	Auria Biobank / University of Turku / Hospital District of Southwest Finland, Turku, Finland	Niina.Pitkanen@tyks.fi	**FinnGen Analysis working group**	**FinnGen Analysis working group**

Samuel Lessard	Translational Sciences, Sanofi R&D, Framingham, MA, USA	samuel.lessard@sanofi.com	**FinnGen Analysis working group**	**FinnGen Analysis working group**
Clément Chatelain	Translational Sciences, Sanofi R&D, Framingham, MA, USA	clement.chatelain@sanofi.com	**FinnGen Analysis working group**	**FinnGen Analysis working group**
Lila Kallio	Auria Biobank / University of Turku / Hospital District of Southwest Finland, Turku, Finland	Lila.Kallio@tyks.fi	**Biobank directors**	**Biobank directors**
Tiina Wahlfors	THL Biobank / Finnish Institute for Health and Welfare (THL), Helsinki, Finland	tiina.wahlfors@thl.fi	**Biobank directors**	**Biobank directors**
Jukka Partanen	Finnish Red Cross Blood Service / Finnish Hematology Registry and Clinical Biobank, Helsinki, Finland	jukka.partanen@veripalvelu.fi	**Biobank directors**	**Biobank directors**
Eero Punkka	Helsinki Biobank / Helsinki University and Hospital District of Helsinki and Uusimaa, Helsinki	eero.punkka@hus.fi	**Biobank directors**	**Biobank directors**
Raisa Serpi	Northern Finland Biobank Borealis / University of Oulu / Northern Ostrobothnia Hospital District, Oulu, Finland	raisa.serpi@ppshp.fi	**Biobank directors**	**Biobank directors**
Sanna Siltanen	Finnish Clinical Biobank Tampere / University of Tampere / Pirkanmaa Hospital District, Tampere, Finland	sanna.siltanen@pshp.fi	**Biobank directors**	**Biobank directors**
Veli-Matti Kosma	Biobank of Eastern Finland / University of Eastern Finland / Northern Savo Hospital District, Kuopio, Finland	veli-matti.kosma@uef.fi	**Biobank directors**	**Biobank directors**
Teijo Kuopio	Central Finland Biobank / University of Jyväskylä / Central Finland Health Care District, Jyväskylä, Finland	teijo.kuopio@ksshp.fi	**Biobank directors**	**Biobank directors**
Anu Jalanko	Institute for Molecular Medicine Finland (FIMM), HiLIFE, University of Helsinki, Helsinki, Finland	anu.jalanko@helsinki.fi	**FinnGen Teams**	**Administration**
Huei-Yi Shen	Institute for Molecular Medicine Finland (FIMM), HiLIFE, University of Helsinki, Helsinki, Finland	huei-yi.shen@helsinki.fi	**FinnGen Teams**	**Administration**
Risto Kajanne	Institute for Molecular Medicine Finland (FIMM), HiLIFE, University of Helsinki, Helsinki, Finland	risto.kajanne@helsinki.fi	**FinnGen Teams**	**Administration**
Mervi Aavikko	Institute for Molecular Medicine Finland (FIMM), HiLIFE, University of Helsinki, Helsinki, Finland	mervi.aavikko@helsinki.fi	**FinnGen Teams**	**Administration**
Helen Cooper	Institute for Molecular Medicine Finland (FIMM), HiLIFE, University of Helsinki, Helsinki, Finland	helen.cooper@helsinki.fi	**FinnGen Teams**	**Administration**
Denise Öller	Institute for Molecular Medicine Finland (FIMM), HiLIFE, University of Helsinki, Helsinki, Finland	denise.oller@helsinki.fi	**FinnGen Teams**	**Administration**
Rasko Leinonen	Institute for Molecular Medicine Finland (FIMM), HiLIFE, University of Helsinki, Helsinki, Finland; European Molecular Biology Laboratory, European Bioinformatics Institute, Cambridge, UK	rasko@ebi.ac.uk	**FinnGen Teams**	**Administration**
Henna Palin	Finnish Clinical Biobank Tampere / University of Tampere / Pirkanmaa Hospital District, Tampere, Finland	henna.palin@pshp.fi	**FinnGen Teams**	**Administration**
Malla-Maria Linna	Helsinki Biobank / Helsinki University and Hospital District of Helsinki and Uusimaa, Helsinki	malla-maria.linna@hus.fi	**FinnGen Teams**	**Administration**
Mitja Kurki	Institute for Molecular Medicine Finland (FIMM), HiLIFE, University of Helsinki, Helsinki, Finland; Broad Institute, Cambridge, MA, United States	mkurki@broadinstitute.org	**FinnGen Teams**	**Analysis**
Juha Karjalainen	Institute for Molecular Medicine Finland (FIMM), HiLIFE, University of Helsinki, Helsinki, Finland	juha.karjalainen@helsinki.fi	**FinnGen Teams**	**Analysis**
Pietro Della Briotta Parolo	Institute for Molecular Medicine Finland (FIMM), HiLIFE, University of Helsinki, Helsinki, Finland	pietro.dellabriottaparolo@helsinki.fi	**FinnGen Teams**	**Analysis**
Arto Lehisto	Institute for Molecular Medicine Finland (FIMM), HiLIFE, University of Helsinki, Helsinki, Finland	arto.lehisto@helsinki.fi	**FinnGen Teams**	**Analysis**
Juha Mehtonen	Institute for Molecular Medicine Finland (FIMM), HiLIFE, University of Helsinki, Helsinki, Finland	juha.mehtonen@helsinki.fi	**FinnGen Teams**	**Analysis**
Wei Zhou	Broad Institute, Cambridge, MA, United States	wzhou@broadinstitute.org	**FinnGen Teams**	**Analysis**
Masahiro Kanai	Broad Institute, Cambridge, MA, United States	mkanai@broadinstitute.org	**FinnGen Teams**	**Analysis**

Mutaamba Maasha	Broad Institute, Cambridge, MA, United States	mmaasha@broadinstitute.org	**FinnGen Teams**	**Analysis**
Zhili Zheng	Broad Institute, Cambridge, MA, United States	zhengzhi@broadinstitute.org	**FinnGen Teams**	**Analysis**
Hannele Laivuori	Institute for Molecular Medicine Finland (FIMM), HiLIFE, University of Helsinki, Helsinki, Finland	hannele.laivuori@helsinki.fi	**FinnGen Teams**	**Clinical Endpoint Development**
Aki Havulinna	Institute for Molecular Medicine Finland (FIMM), HiLIFE, University of Helsinki, Helsinki, Finland; Finnish Institute for Health and Welfare (THL), Helsinki, Finland	aki.havulinna@helsinki.fi	**FinnGen Teams**	**Clinical Endpoint Development**
Susanna Lemmelä	Institute for Molecular Medicine Finland (FIMM), HiLIFE, University of Helsinki, Helsinki, Finland	susanna.lemmela@helsinki.fi	**FinnGen Teams**	**Clinical Endpoint Development**
Tuomo Kiiskinen	Institute for Molecular Medicine Finland (FIMM), HiLIFE, University of Helsinki, Helsinki, Finland	tuomo.kiiskinen@helsinki.fi	**FinnGen Teams**	**Clinical Endpoint Development**
L. Elisa Lahtela	Institute for Molecular Medicine Finland (FIMM), HiLIFE, University of Helsinki, Helsinki, Finland	laura.lahtela@helsinki.fi	**FinnGen Teams**	**Clinical Endpoint Development**
Mari Kaunisto	Institute for Molecular Medicine Finland (FIMM), HiLIFE, University of Helsinki, Helsinki, Finland	mari.kaunisto@helsinki.fi	**FinnGen Teams**	**Communication**
Elina Kilpeläinen	Institute for Molecular Medicine Finland (FIMM), HiLIFE, University of Helsinki, Helsinki, Finland	elina.kilpelainen@helsinki.fi	**FinnGen Teams**	**E-Science**
Timo P. Sipilä	Institute for Molecular Medicine Finland (FIMM), HiLIFE, University of Helsinki, Helsinki, Finland	timo.p.sipila@helsinki.fi	**FinnGen Teams**	**E-Science**
Oluwaseun Alexander Dada	Institute for Molecular Medicine Finland (FIMM), HiLIFE, University of Helsinki, Helsinki, Finland	alexander.dada@helsinki.fi	**FinnGen Teams**	**E-Science**
Awaisa Ghazal	Institute for Molecular Medicine Finland (FIMM), HiLIFE, University of Helsinki, Helsinki, Finland	awaisa.ghazal@helsinki.fi	**FinnGen Teams**	**E-Science**
Anastasia Kytölä	Institute for Molecular Medicine Finland (FIMM), HiLIFE, University of Helsinki, Helsinki, Finland	anastasia.shcherban@helsinki.fi	**FinnGen Teams**	**E-Science**
Rigbe Weldatsadik	Institute for Molecular Medicine Finland (FIMM), HiLIFE, University of Helsinki, Helsinki, Finland	rigbe.weldatsadik@helsinki.fi	**FinnGen Teams**	**E-Science**
Sanni Ruotsalainen	Institute for Molecular Medicine Finland (FIMM), HiLIFE, University of Helsinki, Helsinki, Finland	sanni.ruotsalainen@helsinki.fi	**FinnGen Teams**	**E-Science**
Kati Donner	Institute for Molecular Medicine Finland (FIMM), HiLIFE, University of Helsinki, Helsinki, Finland	kati.donner@helsinki.fi	**FinnGen Teams**	**Genotyping**
Timo P. Sipilä	Institute for Molecular Medicine Finland (FIMM), HiLIFE, University of Helsinki, Helsinki, Finland	timo.p.sipila@helsinki.fi	**FinnGen Teams**	**Genotyping**
Anu Loukola	Helsinki Biobank / Helsinki University and Hospital District of Helsinki and Uusimaa, Helsinki	anu.loukola@hus.fi	**FinnGen Teams**	**Sample Collection Coordination**
Päivi Laiho	THL Biobank / Finnish Institute for Health and Welfare (THL), Helsinki, Finland	paivi.laiho@thl.fi	**FinnGen Teams**	**Sample Logistics**
Tuuli Sistonen	THL Biobank / Finnish Institute for Health and Welfare (THL), Helsinki, Finland	tuuli.sistonen@thl.fi	**FinnGen Teams**	**Sample Logistics**
Essi Kaiharju	THL Biobank / Finnish Institute for Health and Welfare (THL), Helsinki, Finland	essi.kaiharju@thl.fi	**FinnGen Teams**	**Sample Logistics**
Markku Laukkanen	THL Biobank / Finnish Institute for Health and Welfare (THL), Helsinki, Finland	markku.laukkanen@thl.fi	**FinnGen Teams**	**Sample Logistics**
Elina Järvensivu	THL Biobank / Finnish Institute for Health and Welfare (THL), Helsinki, Finland	elina.jarvensivu@thl.fi	**FinnGen Teams**	**Sample Logistics**
Sini Lähteenmäki	THL Biobank / Finnish Institute for Health and Welfare (THL), Helsinki, Finland	sini.lahteenmaki@thl.fi	**FinnGen Teams**	**Sample Logistics**
Lotta Männikkö	THL Biobank / Finnish Institute for Health and Welfare (THL), Helsinki, Finland	lotta.mannikko@thl.fi	**FinnGen Teams**	**Sample Logistics**
Regis Wong	THL Biobank / Finnish Institute for Health and Welfare (THL), Helsinki, Finland	regis.wong@thl.fi	**FinnGen Teams**	**Sample Logistics**
Auli Toivola	THL Biobank / Finnish Institute for Health and Welfare (THL), Helsinki, Finland	auli.toivola@thl.fi	**FinnGen Teams**	**Sample Logistics**
Minna Brunfeldt	THL Biobank / Finnish Institute for Health and Welfare (THL), Helsinki, Finland	minna.brunfeldt@thl.fi	**FinnGen Teams**	**Registry Data Operations**
Hannele Mattsson	THL Biobank / Finnish Institute for Health and Welfare (THL), Helsinki, Finland	hannele.mattsson@thl.fi	**FinnGen Teams**	**Registry Data Operations**

Kati Kristiansson	THL Biobank / Finnish Institute for Health and Welfare (THL), Helsinki, Finland	kati.kristiansson@thl.fi	**FinnGen Teams**	**Registry Data Operations**
Susanna Lemmelä	Institute for Molecular Medicine Finland (FIMM), HiLIFE, University of Helsinki, Helsinki, Finland	susanna.lemmela@helsinki.fi	**FinnGen Teams**	**Registry Data Operations**
Sami Koskelainen	THL Biobank / Finnish Institute for Health and Welfare (THL), Helsinki, Finland	sami.koskelainen@thl.fi	**FinnGen Teams**	**Registry Data Operations**
Tero Hiekkalinna	THL Biobank / Finnish Institute for Health and Welfare (THL), Helsinki, Finland	tero.hiekkalinna@helsinki.fi	**FinnGen Teams**	**Registry Data Operations**
Teemu Paajanen	THL Biobank / Finnish Institute for Health and Welfare (THL), Helsinki, Finland	teemu.paajanen@thl.fi	**FinnGen Teams**	**Registry Data Operations**
Priit Palta	Institute for Molecular Medicine Finland (FIMM), HiLIFE, University of Helsinki, Helsinki, Finland	priit.palta@helsinki.fi	**FinnGen Teams**	**Sequencing Informatics**
Shuang Luo	Institute for Molecular Medicine Finland (FIMM), HiLIFE, University of Helsinki, Helsinki, Finland	shuang.luo@helsinki.fi	**FinnGen Teams**	**Sequencing Informatics**
Tarja Laitinen	Pirkanmaa Hospital District, Tampere, Finland	tarja.laitinen@pshp.fi	**FinnGen Teams**	**Trajectory**
Mary Pat Reeve	Institute for Molecular Medicine Finland (FIMM), HiLIFE, University of Helsinki, Helsinki, Finland	mary.reeve@helsinki.fi	**FinnGen Teams**	**Trajectory**
Shanmukha Sampath Padmanabhuni	Institute for Molecular Medicine Finland (FIMM), HiLIFE, University of Helsinki, Helsinki, Finland	sam.padmanabhuni@helsinki.fi	**FinnGen Teams**	**Trajectory**
Marianna Niemi	University of Tampere, Tampere, Finland	marianna.niemi@tuni.fi	**FinnGen Teams**	**Trajectory**
Harri Siirtola	University of Tampere, Tampere, Finland	harri.siirtola@tuni.fi	**FinnGen Teams**	**Trajectory**
Javier Gracia-Tabuenca	University of Tampere, Tampere, Finland	javier.graciatabuenca@tuni.fi	**FinnGen Teams**	**Trajectory**
Mika Helminen	University of Tampere, Tampere, Finland	mika.helminen@tuni.fi	**FinnGen Teams**	**Trajectory**
Tiina Luukkaala	University of Tampere, Tampere, Finland	tiina.luukkaala@tuni.fi	**FinnGen Teams**	**Trajectory**
Iida Vähätalo	University of Tampere, Tampere, Finland	iida.vahatalo@epshp.fi	**FinnGen Teams**	**Trajectory**
Jyrki Tammerluoto	Institute for Molecular Medicine Finland (FIMM), HiLIFE, University of Helsinki, Helsinki, Finland	jyrki.tammerluoto@helsinki.fi	**FinnGen Teams**	**Data protection officer**
Marco Hautalahti	Finnish Biobank Cooperative - FINBB	marco.hautalahti@finbb.fi	**FinnGen Teams**	**FINBB - Finnish biobank cooperative**
Johanna Mäkelä	Finnish Biobank Cooperative - FINBB	johanna.makela@finbb.fi	**FinnGen Teams**	**FINBB - Finnish biobank cooperative**
Sarah Smith	Finnish Biobank Cooperative - FINBB	sarah.smith@finbb.fi	**FinnGen Teams**	**FINBB - Finnish biobank cooperative**
Tom Southerington	Finnish Biobank Cooperative - FINBB	tom.southerington@finbb.fi	**FinnGen Teams**	**FINBB - Finnish biobank cooperative**
Petri Lehto	Finnish Biobank Cooperative - FINBB	petri.lehto@finbb.fi	**FinnGen Teams**	**FINBB - Finnish biobank cooperative**

9. Estonian Genome Centre Contributors

Full Name	Affiliation	E-mail	Role	Tasks
Andres Metspalu	Estonian Genome Center, Institute of Genomics, University of Tartu, Tartu, Estonia	andres.metspalu@ut.ee	**Estonian Biobank Research Team**	**Data collection, genotyping, QC and imputation**
Lili Milani	Estonian Genome Center, Institute of Genomics, University of Tartu, Tartu, Estonia	lili.milani@ut.ee	**Estonian Biobank Research Team**	**Data collection, genotyping, QC and imputation**
Tõnu Esko	Estonian Genome Center, Institute of Genomics, University of Tartu, Tartu, Estonia	tonu.esko@ut.ee	**Estonian Biobank Research Team**	**Data collection, genotyping, QC and imputation, supervision**
Reedik Mägi	Estonian Genome Center, Institute of Genomics, University of Tartu, Tartu, Estonia	reedik.magi@ut.ee	**Estonian Biobank Research Team**	**Data collection, genotyping, QC and imputation**
Mari Nelis	Estonian Genome Center, Institute of Genomics, University of Tartu, Tartu, Estonia	mari.nelis@ut.ee	**Estonian Biobank Research Team**	**Data collection, genotyping, QC and imputation**
Georgi Hudjashov	Estonian Genome Center, Institute of Genomics, University of Tartu, Tartu, Estonia	georgi.hudjashov@ut.ee	**Estonian Biobank Research Team**	**Data collection, genotyping, QC and imputation**
Erik Abner	Estonian Genome Center, Institute of Genomics, University of Tartu, Tartu, Estonia	erik.abner@ut.ee	**Functional Genomics Team**	**Data analysis**
Urmo Võsa	Estonian Genome Center, Institute of Genomics, University of Tartu, Tartu, Estonia	urmo.vosa@ut.ee	**Functional Genomics Team**	**Data analysis**
Sten Raak	Estonian Genome Center, Institute of Genomics, University of Tartu, Tartu, Estonia	sten.raak@ut.ee	**Functional Genomics Team**	**Data analysis**
